# Autoantibodies neutralizing type I IFNs underlie West Nile virus encephalitis in ∼40% of patients

**DOI:** 10.1084/jem.20230661

**Published:** 2023-06-22

**Authors:** Adrian Gervais, Francesca Rovida, Maria Antonietta Avanzini, Stefania Croce, Astrid Marchal, Shih-Ching Lin, Alessandro Ferrari, Christian W. Thorball, Orianne Constant, Tom Le Voyer, Quentin Philippot, Jérémie Rosain, Micol Angelini, Malena Pérez Lorenzo, Lucy Bizien, Cristian Achille, Francesca Trespidi, Elisa Burdino, Irene Cassaniti, Daniele Lilleri, Chiara Fornara, José Camilla Sammartino, Danilo Cereda, Chiara Marrocu, Antonio Piralla, Chiara Valsecchi, Stefano Ricagno, Paola Cogo, Olaf Neth, Inés Marín-Cruz, Monia Pacenti, Alessandro Sinigaglia, Marta Trevisan, Andrea Volpe, Antonio Marzollo, Francesca Conti, Tiziana Lazzarotto, Andrea Pession, Pierluigi Viale, Jacques Fellay, Stefano Ghirardello, Mélodie Aubart, Valeria Ghisetti, Alessandro Aiuti, Emmanuelle Jouanguy, Paul Bastard, Elena Percivalle, Fausto Baldanti, Anne Puel, Margaret R. MacDonald, Charles M. Rice, Giada Rossini, Kristy O. Murray, Yannick Simonin, Anna Nagy, Luisa Barzon, Laurent Abel, Michael S. Diamond, Aurélie Cobat, Shen-Ying Zhang, Jean-Laurent Casanova, Alessandro Borghesi

**Affiliations:** 1 Laboratory of Human Genetics of Infectious Diseases, Necker Branch, Institut National de la Santé et de la Recherche Médicale (INSERM) U1163, Necker Hospital for Sick Children, Paris, France; 2 Paris Cité University, Imagine Institute, Paris, France; 3 Department of Clinical, Surgical, Diagnostic and Pediatric Sciences, University of Pavia, Pavia, Italy; 4 Microbiology and Virology Unit, San Matteo Research Hospital, Pavia, Italy; 5 Laboratory of Pediatric Hemato-Oncology and Bone Marrow Transplantation, San Matteo Research Hospital, Pavia, Italy; 6 UOSD Cell Factory, San Matteo Research Hospital, Pavia, Italy; 7 Departments of Medicine, Molecular Microbiology, Pathology and Immunology, and The Andrew M. and Jane M. Bursky Center for Human Immunology and Immunotherapy Programs, Washington University School of Medicine, St. Louis, MO, USA; 8 Precision Medicine Unit, Lausanne University Hospital and University of Lausanne, Lausanne, Switzerland; 9 School of Life Sciences, Swiss Federal Institute of Technology, Lausanne, Switzerland; 10 Pathogenesis and Control of Chronic and Emerging Infections, University of Montpellier, INSERM, EFS, Montpellier, France; 11 Study Center for Primary Immunodeficiencies, Necker Hospital for Sick Children, Assistance Publique-Hôpitaux de Paris, Paris, France; 12 Neonatal Intensive Care Unit, San Matteo Research Hospital, Pavia, Italy; 13 Laboratory of Microbiology and Virology, Amedeo di Savoia Hospital, ASL Città di Torino, Turin, Italy; 14 Direzione Generale Welfare, Lombardy, Italy; 15 Department of Biomedical Sciences for Health, Postgraduate School of Public Health, University of Milan, Milan, Italy; 16 Department of Biosciences, University of Milan, Milan, Italy; 17 Institute of Molecular and Translational Cardiology, San Donato Hospital, Milan, Italy; 18 Department of Medicine (DAME), Division of Pediatrics, University of Udine, Udine, Italy; 19 Inborn Errors of Immunity Laboratory, Biomedicine Institute in Seville (IBiS), University of Seville/CSIC, “Red de Investigación Translacional en Infectología Pediátrica”, Seville, Spain; 20 Paediatric Infectious Diseases, Rheumatology and Immunology Unit, Virgen del Rocío University Hospital, Seville, Spain; 21 Microbiology and Virology Unit, Padova University Hospital, Padova, Italy; 22 Department of Molecular Medicine, University of Padova, Padova, Italy; 23 Pediatric Hematology, Oncology and Stem Cell Transplant Division, Padova University Hospital, Padova, Italy; 24 Pediatric Unit, University Hospital of Bologna, Bologna, Italy; 25 Microbiology Unit, IRCCS Azienda Ospedaliero-Universitaria di Bologna, Bologna, Italy; 26 Department of Medical and Surgical Sciences, Section of Microbiology, University of Bologna, Bologna, Italy; 27 Infectious Diseases Unit, University Hospital of Bologna, Bologna, Italy; 28 Pediatric Neurology Department, Necker-Enfants-Malades Hospital, Assistance Publique-Hôpitaux de Paris, Paris, France; 29 Pediatric Immunohematology Unit, IRCCS San Raffaele Scientific Institute, Milan, Italy; 30 San Raffaele Telethon Institute for Gene Therapy (SR-TIGET), IRCCS San Raffaele Scientific Institute, Milan, Italy; 31 Vita-Salute San Raffaele University, Milan, Italy; 32 St. Giles Laboratory of Human Genetics of Infectious Diseases, Rockefeller Branch, The Rockefeller University, New York, NY, USA; 33 Pediatric Hematology-Immunology and Rheumatology Unit, Necker Hospital for Sick Children, Assistante Publique-Hôpitaux de Paris, Paris, France; 34 Laboratory of Virology and Infectious Disease, The Rockefeller University, New York, NY, USA; 35 Department of Pediatrics, Section of Pediatric Tropical Medicine, Center for Human Immunobiology, Baylor College of Medicine and Texas Children’s Hospital, Houston, TX, USA; 36 National Reference Laboratory for Viral Zoonoses, National Public Health Center, Budapest, Hungary; 37 Howard Hughes Medical Institute, New York, NY, USA; 38 Department of Pediatrics, Necker Hospital for Sick Children, Paris, France

## Abstract

Mosquito-borne West Nile virus (WNV) infection is benign in most individuals but can cause encephalitis in <1% of infected individuals. We show that ∼35% of patients hospitalized for WNV disease (WNVD) in six independent cohorts from the EU and USA carry auto-Abs neutralizing IFN-α and/or -ω. The prevalence of these antibodies is highest in patients with encephalitis (∼40%), and that in individuals with silent WNV infection is as low as that in the general population. The odds ratios for WNVD in individuals with these auto-Abs relative to those without them in the general population range from 19.0 (95% CI 15.0–24.0, P value <10^–15^) for auto-Abs neutralizing only 100 pg/ml IFN-α and/or IFN-ω to 127.4 (CI 87.1–186.4, P value <10^–15^) for auto-Abs neutralizing both IFN-α and IFN-ω at a concentration of 10 ng/ml. These antibodies block the protective effect of IFN-α in Vero cells infected with WNV in vitro. Auto-Abs neutralizing IFN-α and/or IFN-ω underlie ∼40% of cases of WNV encephalitis.

## Teaser

Gervais et al. show that auto-Abs neutralizing type I IFNs and pre-existing infection underlie ∼40% of cases of West Nile virus (WNV) encephalitis. Blood and cerebrospinal fluid auto-Abs neutralize the protective activity of type I IFNs against WNV in vitro.

## Introduction

West Nile virus (WNV) is a mosquito-borne neurotropic flavivirus that can trigger life-threatening disease in humans. First identified in the West Nile district of Uganda in 1937 (Smithburn et al., 1940), it emerged as a major global health concern in the 1990s following large outbreaks of severe WNV disease (WNVD) requiring hospitalization, including neuroinvasive disease, in increasingly vast geographic areas worldwide (Petersen et al., 2013a). In recent years, WNV infections have been reported in at least 60 countries across all continents, and the virus is continuing to spread to new areas (Chowdhury and Khan, 2021). WNV is now a leading cause of mosquito-borne disease globally, with the number of infections estimated in the millions, tens of thousands of reported cases of neuroinvasive disease, and thousands of deaths reported in countries with active surveillance systems over the last 20 yr (Petersen et al., 2013a; Petersen et al., 2013b; Centers for Disease Control and Prevention, 2023; European Centre for Disease Prevention and Control. 2023). Nevertheless, most infected individuals remain asymptomatic, with only ∼20% reporting a self-limited, febrile illness (WNV fever, WNVF), and <1% requiring hospitalization for neuroinvasive disease, including encephalitis (50–70%), meningitis (15–35%), and acute flaccid paralysis (3–20%), resulting in a mortality of about 5–20% (Lindsey et al., 2010). This immense interindividual clinical variability has remained largely unexplained (Barzon et al., 2022). Epidemiologically, age is the strongest known predictor of neuroinvasive disease and death (Patel et al., 2015; Solomon, 2004). The seroprevalence of IgG or IgM against this virus is similar in all age groups (Mostashari et al., 2001; Tsai et al., 1998), but the risk of severe disease, particularly neuroinvasive disease, is about 16 times higher in those over 65 yr than in younger individuals (Carson et al., 2012), and the risk of death is about 30–45 times higher in those over 70 yr of age than in younger individuals (Kopel et al., 2011; Lindsey et al., 2012). Being male also increases the risk of neuroinvasive disease, albeit to a lesser extent (odds ratio [OR] = 1.3–1.6; Sutinen et al., 2022). Some comorbid conditions and immunosuppression also lead to a modest increase in the risk of neuroinvasive disease and death (Nash et al., 2001; Murray et al., 2006; Murray et al., 2009; Sutinen et al., 2022).

Little is known about the molecular and cellular determinants of WNVD in natural conditions of infection. Homozygosity for the CCR5Δ32 deletion at the *CCR5* locus significantly increases the risk of symptomatic infection (OR = 4.5, 95% confidence interval [CI] = 2.2–9.4) and death (OR = 6.6, 95% CI: 1.2–37; Glass et al., 2006). More is known about the molecular basis of immunity to experimental WNV infection. Type I interferons (IFNs) confer cell-intrinsic protection against WNV replication in vitro in human cells, including dermal fibroblasts and various cell lines (Schoggins et al., 2011). They also protect mice in vivo (Suthar et al., 2013; Samuel and Diamond, 2005). These findings suggest that insufficient type I IFN immunity might underlie life-threatening WNVD, in at least some patients. Moreover, the higher risk of WNVD in men over the age of 65 yr is reminiscent of the situation for critical coronavirus disease 2019 (COVID-19) pneumonia (O’Driscoll et al., 2021), for which ∼15% of cases are due to pre-existing circulating auto-Abs neutralizing type I IFNs (Bastard et al., 2020; Bastard et al., 2021a; Manry et al., 2022). Serum or plasma from patients with these antibodies, tested at a 1/10 dilution, neutralizes low (100 pg/ml) or high (10 ng/ml) concentrations of IFN-α and/or IFN-ω. These auto-Abs have a prevalence among individuals under the age of 65 yr in the general population of ∼0.3% (Abs neutralizing high concentrations) and ∼1% (Abs neutralizing low concentrations); this prevalence increases sharply after the age of 70 yr to ∼4 and ∼7%, respectively (Bastard et al., 2021a; Bastard et al., 2022; Puel et al., 2022; Zhang et al., 2022a). Auto-Abs neutralizing IFN-β are rarer in the general population and their prevalence does not appear to increase with age (Bastard et al., 2021a). In patients with severe COVID-19 pneumonia carrying auto-Abs against type I IFNs, these antibodies precede infection and are causal for the disease (Manry et al., 2022; Zhang et al., 2022a; Casanova and Abel, 2021; Casanova and Abel, 2022). They also favor varicella zoster virus disease, particularly in patients hospitalized for COVID-19 (Vallbracht et al., 1981; Pozzetto et al., 1984; Walter et al., 2015; Busnadiego et al., 2022), and underlie ∼5% of cases of critical influenza pneumonia (Zhang et al., 2022b) and ∼25% of hospitalizations for Middle East respiratory syndrome (MERS) pneumonia (Alotaibi et al., 2023; Hale, 2023; Puel et al., 2022). Moreover, three of eight patients with adverse reactions (including encephalitis) to the live attenuated virus vaccine against yellow fever virus (YFV-17D), another flavivirus, also carried these neutralizing auto-Abs (Bastard et al., 2021b). In this context, we hypothesized that circulating auto-Abs neutralizing type I IFNs might underlie WNVD in some patients.

## Results

### Six cohorts of patients with WNV infection

**Figure 1.**
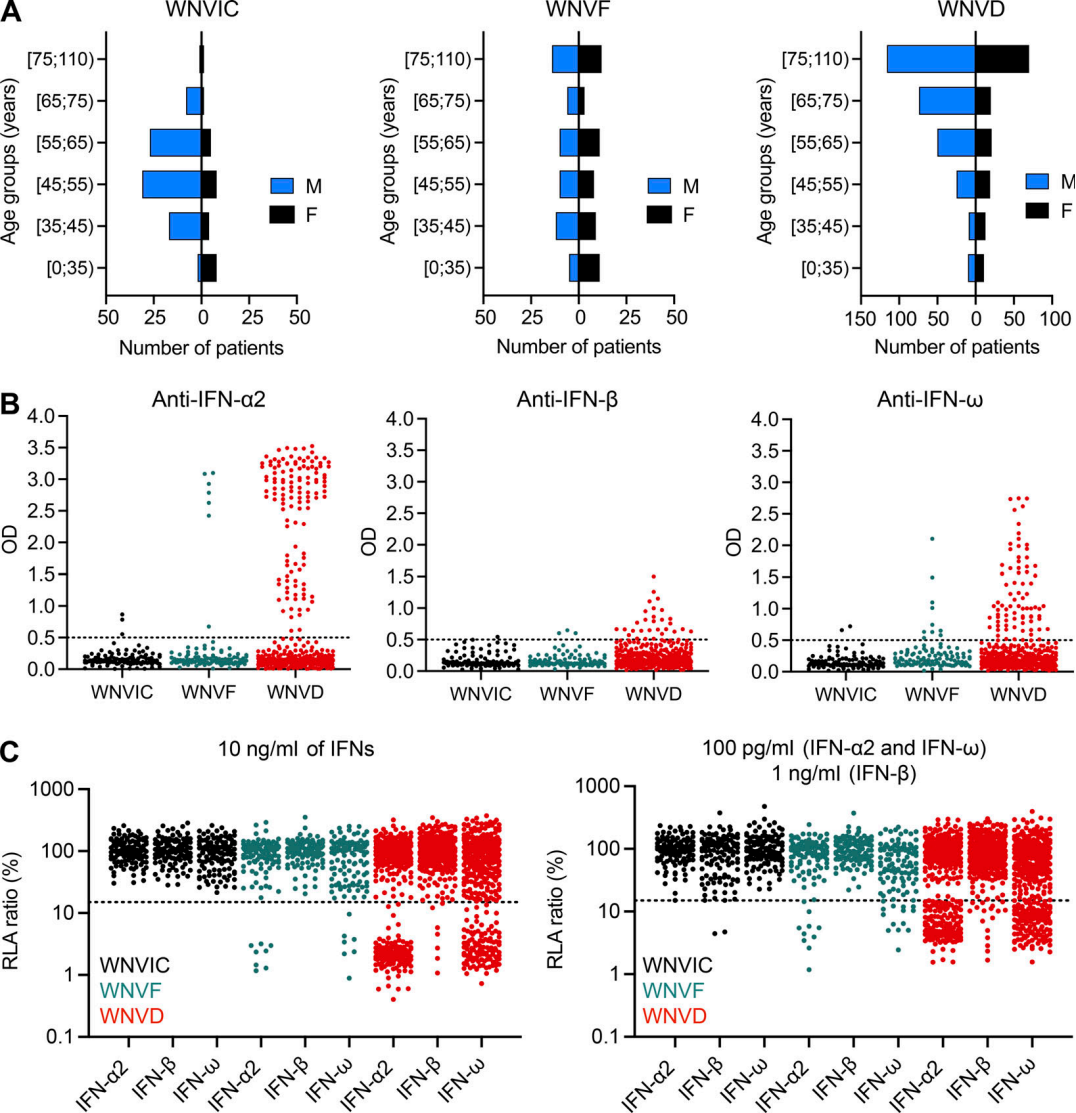
**Auto-Abs neutralizing type I IFNs in individuals infected with WNV. (A)** Age and sex distribution of individuals in the WNVIC, WNVF, and WNVD groups. **(B)** Detection of auto-Abs against IFN-α2, IFN-β, and IFN-ω by ELISA. An OD > 0.5 (dotted line) was considered to correspond to positive samples based on the signal typically observed for serum/plasma from healthy donors. Each sample was tested once. **(C)** Luciferase-based neutralization assay to detect auto-Abs neutralizing 10 ng/ml IFN-α2, IFN-ω, or IFN-β (left panel) and 100 pg/ml IFN-α2 or IFN-ω, or 1 ng/ml IFN-β (right panel). Plasma samples from WNVIC (black), patients with WNVF (dark cyan), and patients with WNVD (red) were diluted 1:10. HEK293T cells were transfected with (1) a plasmid containing the firefly luciferase gene under the control of an IFN-sensitive response element (ISRE)-containing promotor and (2) a plasmid containing the *Renilla* luciferase gene. The cells were then treated with type I IFNs, and relative luciferase activity (RLA) was calculated by normalizing firefly luciferase activity against *Renilla* luciferase activity. An RLA <15% of the value for the mock treatment was considered to correspond to neutralizing activity (dotted line; Bastard et al., 2021a). Each sample was tested once.

**Figure S1.**
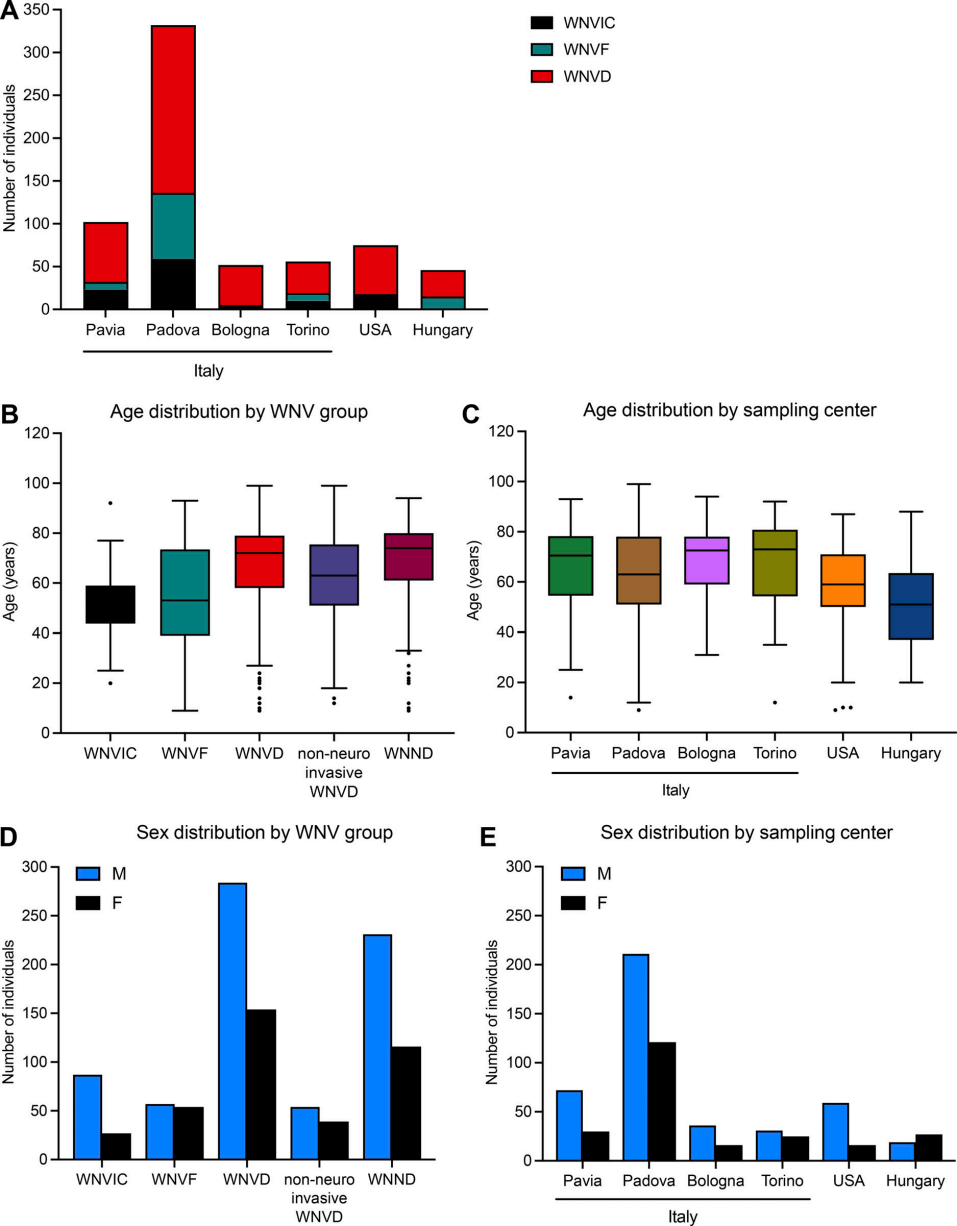
**Detailed demographic characteristics of the individuals enrolled in the study. (A)** Number of individuals of each WNV group enrolled at each center. **(B)** Age distribution of the individuals enrolled, by the WNV group. **(C)** Age distribution of the individuals enrolled, by the center. **(D)** Sex distribution of the individuals enrolled, by the WNV group. **(E)** Sex distribution of the individuals enrolled, by the center. WNND: WNV neuroinvasive disease, a subgroup of WNVD.

We studied 441 subjects hospitalized for WNVD (i.e., with life-threatening disease) from Italy (four cohorts, 353 individuals), the USA (57 individuals), and Hungary (31 individuals). There were 348 patients with confirmed neurological diseases—encephalitis (222 cases), meningitis (87 cases), acute flaccid paralysis (8 cases), and other unspecified neurological syndromes (31 cases)—and 93 patients without clinical evidence of neuroinvasive disease. We also enrolled 108 patients with WNVF managed as outpatients in Italy and Hungary. Finally, we enrolled 114 individuals in Italy and the USA with recent asymptomatic or paucisymptomatic WNV infection (WNV-infected controls, WNVIC) diagnosed on the basis of the detection of WNV RNA in a nucleic acid amplification test (NAAT) in blood, in most cases (98%) performed at the time of blood donation (Fig. 1 A). The individuals included in the Italian cohorts were enrolled at three centers in northern Italy, in Pavia (102 subjects), Padova (332 subjects), and Torino (56 subjects), during the 2018 and 2022 outbreaks, and a fourth center in Bologna during the 2022 outbreak (52 subjects); the Hungarian samples were collected during the 2018 outbreak (46 subjects); and the American samples were collected from participants of the Houston West Nile Cohort (Murray et al., 2009) between 2002 and 2018 (75 subjects; Fig. S1 A). For all the individuals enrolled, there was clear evidence of WNV infection documented by the serological demonstration of WNV-specific IgM or seroconversion to IgG, WNV neutralization assays (Percivalle et al., 2020), and/or RT-PCR on serum, plasma, or cerebrospinal fluid (CSF) samples collected between 2002 and 2022. The mean age (standard deviation, SD) of patients with WNVD was 67 yr (16 yr), and the age of the patients in this group ranged from 9 to 99 yr. The mean age was similar in the subgroup of patients with neuroinvasive disease (69 yr [15 yr]). The mean age (SD) for the patients with WNVF was 55 yr (20), with the patient age in this group ranging from 9 to 93 yr; and the mean age (SD) in the WNVIC group was 50 yr (12), with the age of these individuals ranging from 20 to 92 yr (Fig. S1 B). The mean age (SD) of patients with WNVD was higher in the four Italian cohorts (73 yr [13] in Pavia, 68 yr [17] in Padova, 71 yr [11] in Bologna, 71 yr [15] in Torino; 70 yr [15] overall) than in the American (59 yr [17]) and Hungarian (55 yr [20]) cohorts (P value < 0.001; Fig. S1 C). The proportion of male subjects was 65% for all patients with WNVD (67% among neuroinvasive disease cases), 51% for patients with WNVF, and 76% for WNVIC (P < 0.001; Fig. 1 A and Fig. S1 D). Mortality was assessed based on vital status data, which were available for 314/348 patients with neuroinvasive disease. Mortality was 8% for the entire population of patients with neuroinvasive disease, 9% for those with encephalitis, 5% for those with meningitis, and 9% for patients with unspecified neurological syndrome (Chancey et al., 2015). No deaths were reported among patients with acute flaccid paralysis or patients hospitalized without evidence of neuroinvasive disease (Table 1).

**Table 1.** Demographic and clinical characteristics of the study population in the three WNV clinical groups and the two subgroups of WNVD

Characteristic	WNVIC *n* = 114^a^	WNVF *n* = 108^a^	WNVD *n* = 441^a^	Subgroups of WNVD	
				**Non-neuroinvasive WNVD** ***n* = 93** ^a^	**Neuroinvasive WNVD** ***n* = 348** ^a^
Age (yrs)	50.5 (12.0)	55.4 (20.1)	67.5 (16.5)	60.7 (18.5)	69.3 (15.4)
**Sex**					
F	27 (23.7%)	53 (49.1%)	155 (35.1%)	39 (41.9%)	116 (33.3%)
M	87 (76.3%)	55 (50.9%)	286 (64.9%)	54 (58.1%)	232 (66.7%)
Mortality	0/114	0/108	25/400 (6.3%)	0/86	25/314 (8.0%)
Unknown	0	0	41	7	34
**Recruitment center**					
Italy (PV)	23 (20.2%)	9 (8.3%)	70 (15.9%)	9 (9.7%)	61 (17.5%)
Italy (PD)	59 (51.7%)	77 (71.3%)	196 (44.4%)	71 (76.3%)	125 (35.9%)
Italy (BO)	4 (3.5%)	1 (0.9%)	47 (10.7%)	5 (5.4%)	42 (12.1%)
Italy (TO)	10 (8.8%)	6 (5.6%)	40 (9.1%)	2 (2.1%)	38 (10.9%)
USA	18 (15.8%)	0 (0.0%)	57 (12.9%)	0 (0.0%)	57 (16.4%)
Hungary	0 (0.0%)	15 (13.9%)	31 (7.0%)	6 (6.5%)	25 (7.2%)
**Year of recruitment**					
Before 2018	14 (12.3%)	0 (0.0%)	57 (12.9%)	0 (0.0%)	57 (16.4%)
2018–2019	45 (39.5%)	62 (57.4%)	169 (38.3%)	60 (64.5%)	109 (31.3%)
2022	55 (48.2%)	46 (42.6%)	215 (48.8%)	33 (35.5%)	182 (52.3%)

PV: Pavia; PD: Padova; BO: Bologna; TO: Torino.

a Mean (SD); or counts (frequency, %).

### Auto-Abs against IFN-α2, -β, and/or -ω in patients with WNVD

**Figure S2.**
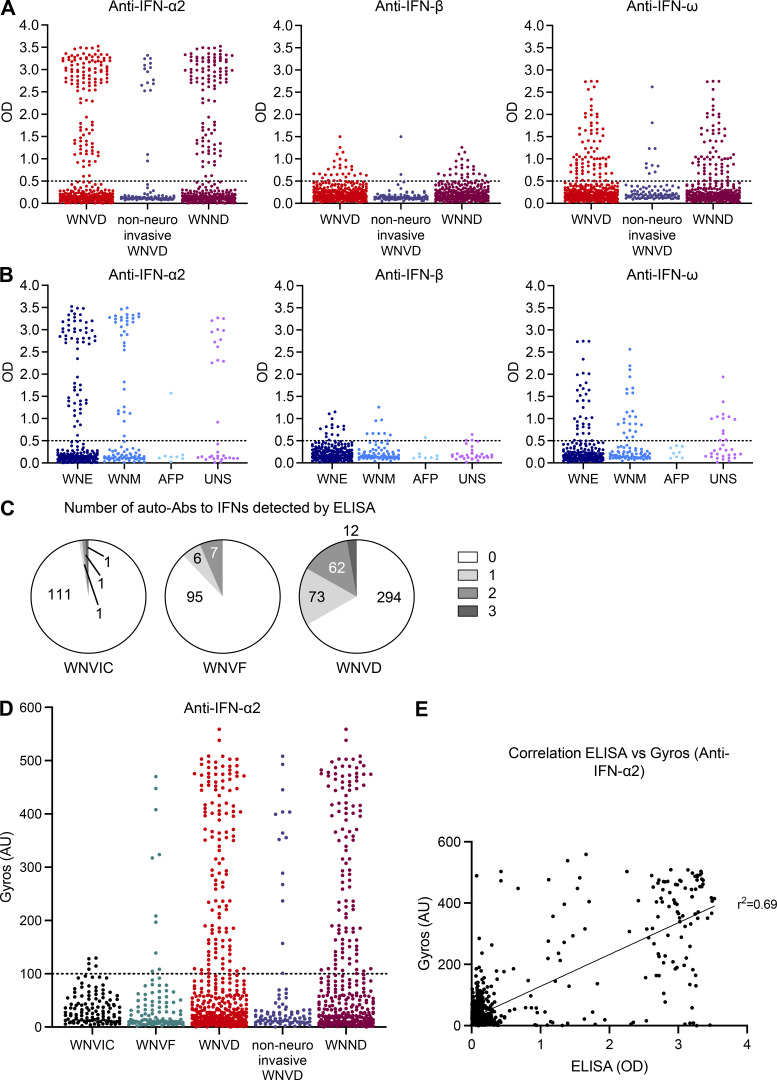
**Detection of auto-Abs against type I IFNs by ELISA and Gyros. (A)** Detection, by ELISA, of auto-Abs against IFN-α2, IFN-β, and IFN-ω for the WNVD group and the two subgroups of WNVD patients: non-neuroinvasive WNVD and neuroinvasive WNVD (WNND). **(B)** Detection, by ELISA, of auto-Abs against IFN-α2, IFN-β, and IFN-ω in subgroups of WNND patients: WNV encephalitis (WNE), WNV meningitis (WNM), acute flaccid paralysis (AFP), and unspecified neurological syndrome (UNS). **(C)** Number of auto-Abs detected by ELISA for the WNVIC, WNVF, and WNVD groups. **(D)** Detection, by Gyros, of auto-Abs against IFN-α2 for the WNVIC, WNVF, and WNVD groups and for patients hospitalized with WNND and without neuroinvasive disease, the two main subgroups of WNVD. **(E)** Correlation between the detection of auto-Abs against IFN-α2 by ELISA and by Gyros. In all the experiments each sample was tested once.

We used an enzyme-linked immunosorbent assay (ELISA) to screen serum or plasma samples from the 663 individuals enrolled in the three groups of individuals infected with WNV (WNVIC [114], WNVF [108], and WNVD [441]) for circulating IgG auto-Abs against type I IFNs. For patients with symptoms, blood samples were taken at a mean of 15 d (range: 0–180 d) after clinical disease onset for the Italian cohorts, 16 d (range: 0–84 d) after clinical disease onset for the Hungarian cohort, and during convalescence, a mean of 618 d (range: 133–4,334 d) after disease onset, for the US cohort. We found high levels, defined as an optical density (OD) >0.5 on ELISA, of auto-Abs against IFN-α2, IFN-β, and/or IFN-ω in 147/441 (33%) patients with WNVD (126/348 [36%] with neuroinvasive disease and 21/93 [23%] patients hospitalized without documented neurological disease). Auto-Abs against at least one type I IFN were detected in 31% of encephalitis cases, 46% of meningitis cases, 52% of cases of unspecified neurological syndrome, and in one patient with acute flaccid paralysis. We also found auto-Abs against at least one type I IFN in 13/108 (12%) outpatients with WNVF and 3/114 (3%) samples from WNVIC (Fig. 1 B; and Fig. S2, A–C). We found that 17% of the patients with WNVD had one type of auto-Ab against IFN-α2 (53/441, 12%), IFN-β (7/441, 2%), or IFN-ω (13/441, 3%), whereas 14% had two auto-Abs directed against IFN-α2 and IFN-β (8/441, 2%), IFN-α2 and IFN-ω (52/441, 12%), or IFN-β and IFN-ω (2/441, <1%) and 12/441 (3%) had auto-Abs against the three type I IFNs tested. A similar distribution was observed in all the neuroinvasive disease subgroups. We found that 16% of patients with severe WNVD without evidence of neuroinvasive disease had only one type of auto-Ab against IFN-α2 (10/93, 11%), IFN-β (1/93, 1%), or IFN-ω (4/93, 4%), accounting for most of the individuals with auto-Abs within this group (15/21, 71%), whereas only 5% of the patients in this group had a combination of auto-Abs against IFN-α2 and IFN-ω, and only one patient (1%) had three auto-Abs directed against the three type I IFNs (Fig. S2 C). We confirmed the ELISA results by testing samples from the patients and WNVIC from all the cohorts with Gyros technology for auto-Abs against IFN-α2 (Fig. S2 D; Honda et al., 2005). The two assays gave almost identical proportions of auto-Ab-positive (>100 AU) individuals, and the concordance between Gyros and ELISA results was high in all the cohorts and WNV-infected groups tested (Fig. S2 E). Overall, our data indicate that ∼35% of all hospitalized WNVD cases, including ∼40% of patients with neuroinvasive disease, had detectable auto-Abs against at least one type I IFN, a prevalence much higher than that reported in the general population (∼1%) and in patients with life-threatening COVID-19 (∼15%; Bastard et al., 2020; Bastard et al., 2021a), influenza pneumonia (∼5%; Zhang et al., 2022b), and MERS pneumonia (∼25%; Alotaibi et al., 2023), but similar to that found in a very small series of patients with severe YFV-17D live-attenuated vaccine disease (three of eight patients; Bastard et al., 2021b; Puel et al., 2022).

### Auto-Abs neutralizing IFN-α2, -β, and/or -ω in patients with WNVD

**Figure 2.**
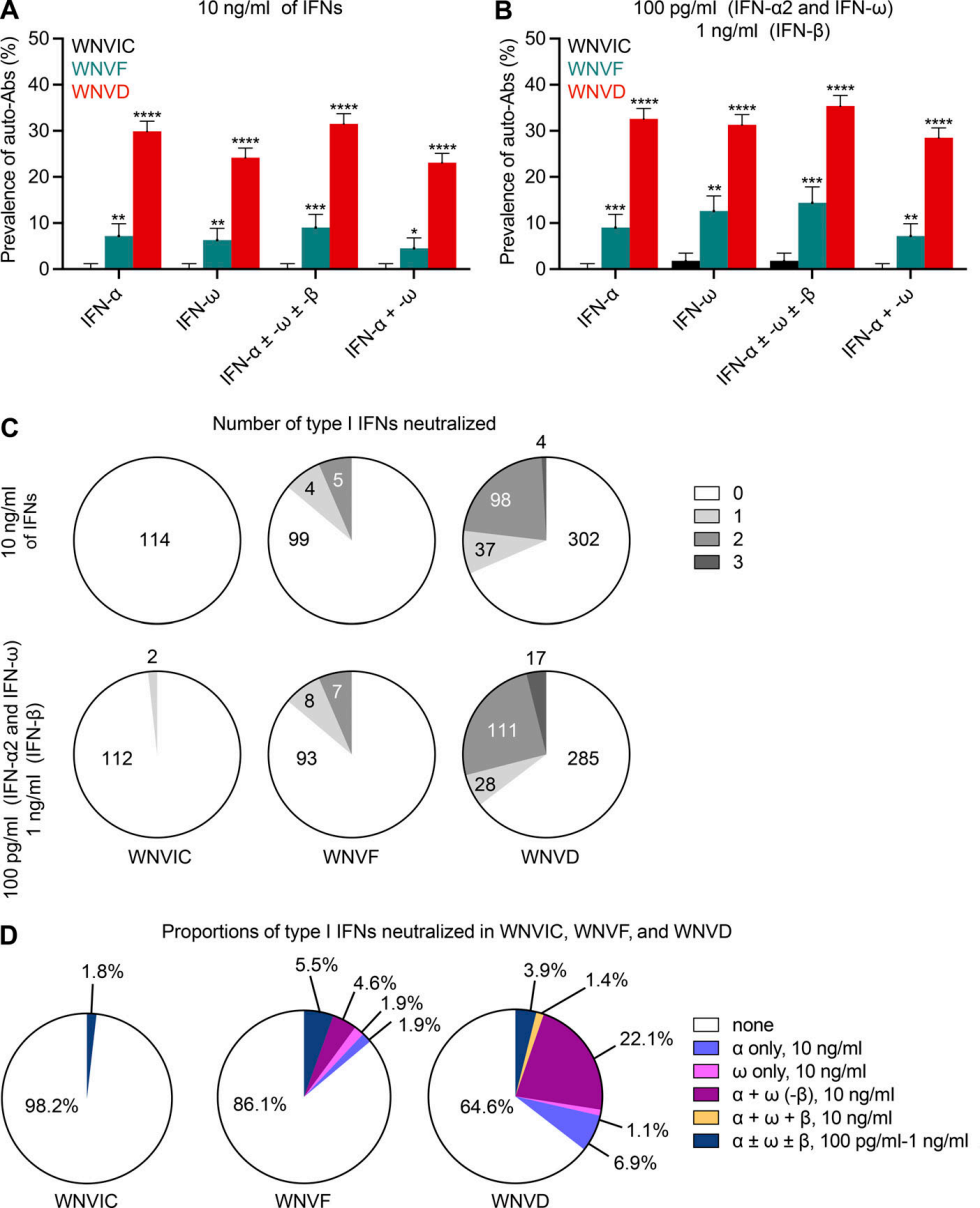
**Proportions of patients with auto-Abs neutralizing type I IFNs. (A and B)** Proportions of individuals with auto-Abs neutralizing type I IFNs at a concentration of 10 ng/ml (A) or 100 pg/ml (B) in the three groups of WNV-infected individuals (WNVIC, WNVF, and WNVD), as determined with the luciferase-based neutralization assay. IFN-α, auto-Abs neutralizing IFN-α2 (regardless of their effects on other IFNs); IFN-ω, auto-Abs neutralizing IFN-ω (regardless of their effects on other IFNs); IFN-α ± ω ± β, auto-Abs neutralizing IFN-α2 and/or IFN-ω and/or IFN-β; IFN-α + ω, auto-Abs neutralizing both IFN-α2 and IFN-ω; *P *<* 0.05, **P *<* 0.01, ***P *<* 0.001, ****P *<* 0.0001. **(C)** The number of type I IFNs neutralized in the three groups of WNV-infected individuals (WNVIC, WNVF, and WNVD) as determined with the luciferase-based neutralization assay. **(D)** Proportion of type I IFNs neutralized in the three groups of WNV-infected individuals (WNVIC, WNVF, and WNVD) according to the nature and combination of auto-Abs.

**Figure S3.**
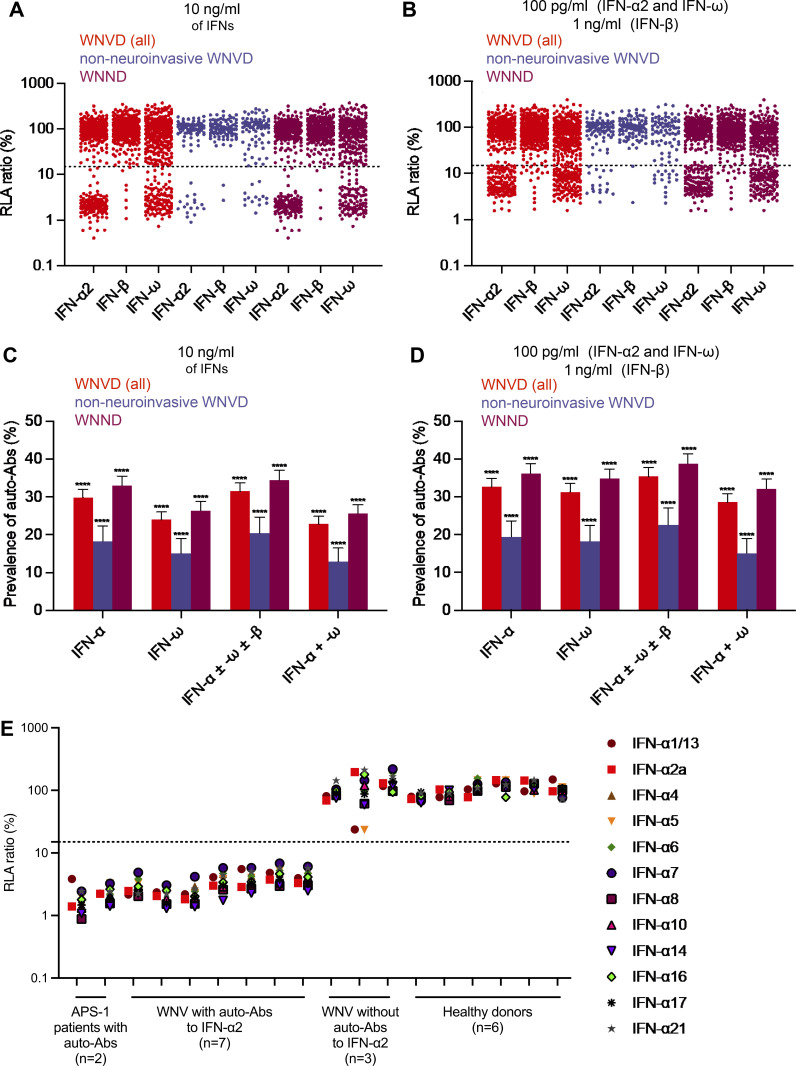
**Detection of auto-Abs neutralizing type I IFNs in the WNVD subgroups and neutralization of 12 IFN-α subtypes. (A and B)** Luciferase-based neutralization assays to detect auto-Abs neutralizing 10 ng/ml IFN-α2, IFN-ω, or IFN-β (A) and 100 pg/ml of IFN-α2 or IFN-ω, or 1 ng/ml of IFN-β (B) in the WNVD group and the two WNVD subgroups of patients hospitalized with neuroinvasive WNVD (WNND) and without neuroinvasive disease. Values below 15% are considered to be associated with the presence of neutralizing auto-Abs. **(C and D)** Proportions of individuals with auto-Abs neutralizing type I IFNs at a concentration of 10 ng/ml (C) or 100 pg/ml for IFN-α2 or IFN-ω, or 1 ng/ml for IFN-β (D) in the WNVD group and the two WNVD subgroups of patients hospitalized with WNND and without neuroinvasive disease; ****P < 10^−4^. **(E)** Neutralization of different subtypes of IFN-α by serum or plasma from individuals with auto-Abs neutralizing IFN-α2. Patients with autoimmune polyendocrine syndrome type I (APS-1), individuals with WNVD without auto-Abs, and healthy controls are shown for comparison. Each sample was tested once.

Using a previously described luciferase-based neutralization assay (Bastard et al., 2021a), we tested 1:10 dilutions of serum or plasma samples from all enrolled subjects, with or without auto-Ab detection by ELISA or Gyros, for the neutralization of high (10 ng/ml) or low (100 pg/ml) concentrations of non-glycosylated IFN-α2 and/or IFN-ω, and/or high (10 ng/ml) or intermediate (1 ng/ml) concentrations of glycosylated IFN-β. We found no auto-Abs neutralizing high or intermediate concentrations (10 ng/ml) of IFN-α2 and/or IFN-β and/or IFN-ω in WNVIC. The prevalence of auto-Abs increased significantly with disease severity, from 9/108 (8%) in patients with WNVF (P = 0.001 versus WNVIC) to 137/441 (31%) in patients with WNVD (P < 10^−15^), 120/348 (34%) in the subgroup of WNVD patients with neuroinvasive disease (P < 10^−15^), and 10/25 (40%) in the patients who died (P = 6 × 10^−9^; Fig. 1 C; Fig. 2 A; and Fig. S3, A and C). At the more physiological concentration of 100 pg/ml, we found that 2 of the 114 WNVIC (2%), 15 of the 108 (14%) patients with WNVF (P = 0.0007 versus WNVIC), 156 of the 441 (35%) individuals with severe WNVD (P < 10^−15^), including 135 of the 348 (39%) individuals with neuroinvasive disease (P < 10^−15^) and 11 of the 25 (44%) patients who died (P = 4.5 × 10^−8^) had antibodies neutralizing IFN-α2 and/or IFN-ω (Fig. 1 C; Fig. 2 B; and Fig. S3, B and D). All the samples from individuals with auto-Abs neutralizing intermediate (1 ng/ml) concentrations of IFN-β also neutralized low concentrations of IFN-α2 and/or IFN-ω. We therefore focused our subsequent analyses on individuals with auto-Abs neutralizing 100 pg/ml IFN-α2 and/or IFN-ω. For the WNVD group, we found that at this concentration, 16/441 (4%) had antibodies neutralizing IFN-α2 only (14/348 [4%] in the subgroup of WNVD patients with neuroinvasive disease), 12/441 (3%) had antibodies neutralizing IFN-ω only (9/348 [3%] of patients with neuroinvasive disease), and 127/441 (29%) had antibodies neutralizing both IFN-α2 and IFN-ω (112/348 [32%] of patients with neuroinvasive disease), with 17/127 (13%; 16/112 [13%] patients with neuroinvasive disease) patients also having antibodies capable of neutralizing IFN-β (Table 2; and Fig. 2, C and D).

**Table 2.** Type I IFN-neutralizing auto-Abs in the study population

**Characteristic**	**Auto-Ab** ^**+**^ **, ** ***n*** ** = 173** ^a^	**No auto-Ab, ** ***n*** ** = 490** ^a^
Age (yrs)	71.1 (14.5)	59.6 (18.0)
≤40	8 (9.4%)	77 (90.6%)
(40–65]	36 (14.2%)	218 (85.8%)
>65	129 (39.8%)	195 (60.2%)
**Sex**		
F	49 (28.3%)	186 (38.0%)
M	124 (71.7%)	304 (62.0%)
**WNV groups**		
WNVD	156 (35.4%)	285 (64.6%)
WNVF	15 (13.9%)	93 (86.1%)
WNVIC	2 (1.8%)	112 (98.2%)
**Subgroups of WNVD**		
Non-neuroinvasive WNVD	21 (22.6%)	72 (77.4%)
**Neuroinvasive WNVD**		
All	135 (38.8%)	213 (61.2%)
WNE	76 (34.2%)	146 (65.8%)
WNM	41 (47.1%)	46 (52.9%)
AFP	2 (25.0%)	6 (75.0%)
UNS	16 (51.6%)	15 (48.4%)

^WNE: WNV encephalitis; WNM: WNV meningitis; AFP: acute flaccid paralysis; UNS: unspecified neurological syndrome.^

a Mean (SD), counts or frequency (%).

### The detection of auto-Abs neutralizing IFN-α2, -β, and/or -ω is highly correlated with ELISA and Gyros results

**Figure S4.**
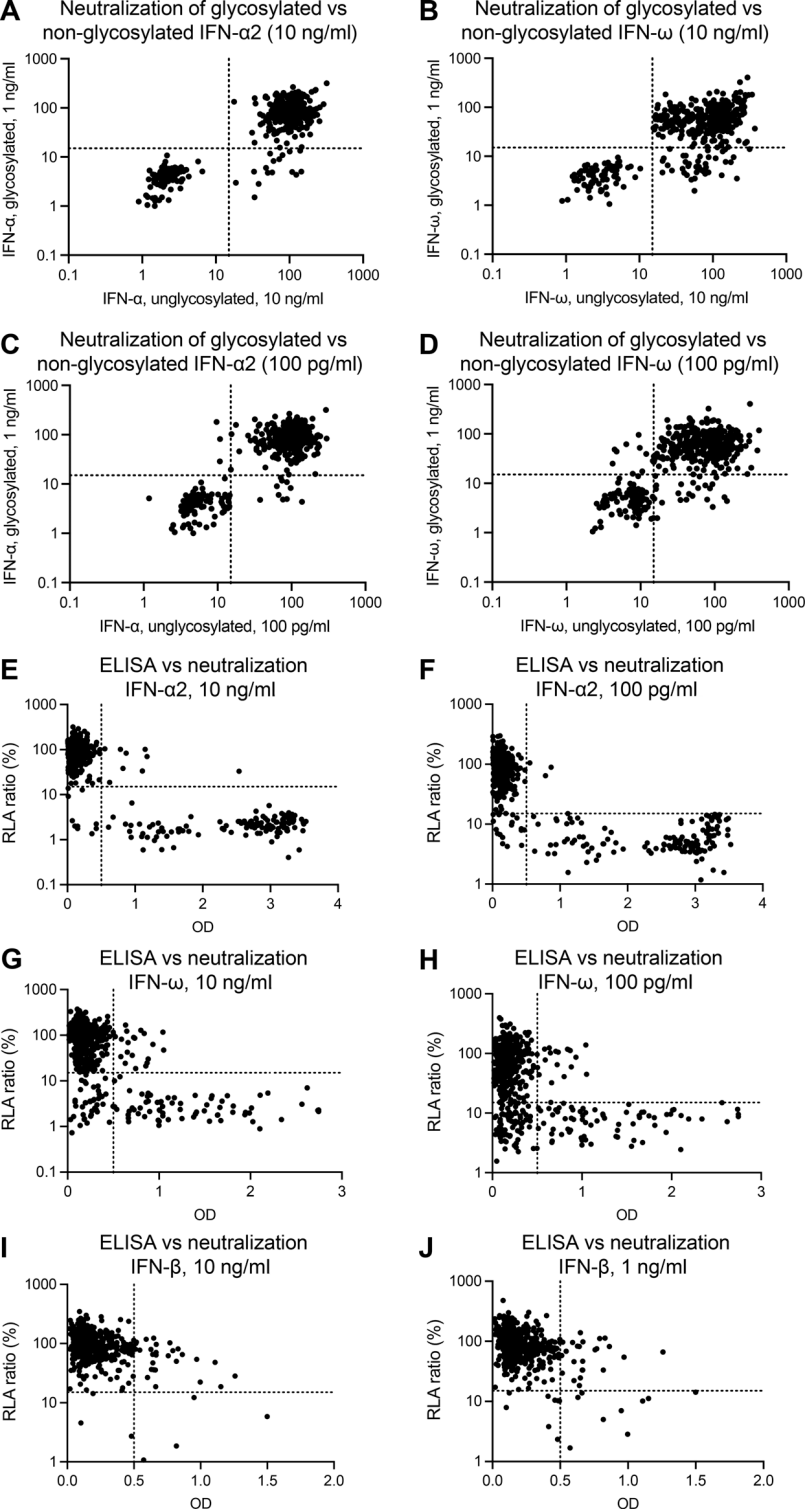
**Correlation between the results of luciferase-based neutralization assays with the glycosylated and non-glycosylated IFNs and between the results of neutralization assays and ELISA for detecting type I IFNs. (A–D)** Correlation between neutralization of 10 ng/ml non-glycosylated IFN-α2 and 1 ng/ml glycosylated IFN-α2 (A), neutralization of 10 ng/ml non-glycosylated IFN-ω and 1 ng/ml glycosylated IFN-ω (B), neutralization of 100 pg/ml non-glycosylated IFN-α2 and 1 ng/ml glycosylated IFN-α2 (C), neutralization of 100 pg/ml non-glycosylated IFN-ω and 1 ng/ml glycosylated IFN-ω (D). **(E)** Detection of auto-Abs against IFN-α2 by ELISA versus neutralizing capacity for a concentration of 10 ng/ml. **(F)** Detection of auto-Abs against IFN-α2 by ELISA versus neutralizing capacity for a concentration of 100 pg/ml. **(G)** Detection of auto-Abs against IFN-ω by ELISA versus neutralizing capacity for a concentration of 10 ng/ml. **(H)** Detection of auto-Abs against IFN-ω by ELISA versus neutralizing capacity for a concentration of 100 pg/ml. **(I)** Detection of auto-Abs against IFN-β by ELISA versus neutralizing capacity for a concentration of 10 ng/ml. **(J)** Detection of auto-Abs against IFN-β by ELISA versus neutralizing capacity for a concentration of 1 g/ml. The relative luciferase activity (RLA) was calculated by normalizing firefly luciferase activity against *Renilla* luciferase activity. An RLA <15% of the value for the mock treatment was considered to correspond to neutralizing activity.

As previously documented for patients with COVID-19 influenza (Bastard et al., 2020; Bastard et al., 2021a), we showed that five randomly selected samples neutralizing IFN-α2 neutralized the other 11 subtypes of IFN-α2 (Fig. S3 E). The 13 *IFNA* loci encode only 12 different proteins because the products of *IFNA1* and *IFNA13* are identical. We also tested 512 serum or plasma samples from four cohorts (Pavia, Padova, Bologna, and Hungary) for the neutralization of 1 ng/ml glycosylated IFN-α2 and IFN-ω, the lowest concentration validated for these experiments to date. We observed very strong concordance between the results obtained for the neutralization of glycosylated and non-glycosylated type I IFNs. All samples neutralizing 10 ng/ml non-glycosylated IFN-α2 and IFN-ω also neutralized the corresponding glycosylated type I IFN at a concentration of 1 ng/ml (Fig. S4, A and B). Only 3.5 and 8.2% of samples neutralizing the glycosylated forms of IFN-α2 and IFN-ω, respectively, did not neutralize the corresponding non-glycosylated IFNs at a concentration of 10 ng/ml, and only 2.1 and 4.7% of samples neutralizing 1 ng/ml glycosylated IFN-α2 and IFN-ω, respectively, did not neutralize the non-glycosylated forms at a concentration of 100 pg/ml, suggesting a higher affinity of these auto-Abs for the glycosylated forms of type I IFNs in these subjects (Fig. S4, A–D). We also found that 1.2 and 2.7% of samples neutralizing 100 pg/ml non-glycosylated IFN-α2 and IFN-ω, respectively, did not neutralize the corresponding glycosylated IFN at a concentration of 1 ng/ml, an observation probably explained by the higher concentration of type I IFNs used in experiments with the glycosylated forms (Fig. S4, C and D). All samples from patients with very high levels (OD >1.5) of IgG auto-Abs on ELISA neutralized the corresponding cytokine, and only a few samples with OD values between 0.5 and 1.5 did not (0–3% of all samples tested for the three cytokines), demonstrating the high specificity of ELISA at both the 0.5 and 1.5 thresholds for detecting these auto-Abs in a first-line diagnostic screen (Bastard et al., 2021a). Similarly, only a small proportion of samples with OD values < 0.5 for auto-Abs against IFN-α2, IFN-β, or IFN-ω neutralized the corresponding cytokine (Fig. S4, E–J), demonstrating the greater sensitivity of neutralization assays over ELISA for detecting such auto-Abs and suggesting that neutralization assays may be an effective way of excluding the presence of pathogenic auto-Abs against type I IFNs in plasma or serum testing negative by ELISA or Gyros. Overall, we demonstrated the presence of auto-Abs neutralizing at least IFN-α2 or IFN-ω in 35% (for 100 pg/ml) and 30% (for 10 ng/ml) of individuals hospitalized for WNVD. No patient had antibodies neutralizing IFN-β only, and 11% of WNVD patients with auto-Abs neutralizing 100 pg/ml and 11% of those with auto-Abs neutralizing 10 ng/ml IFN-α2 or IFN-ω also neutralized IFN-β.

### Higher prevalence of auto-Abs in male individuals and the over-65s

**Figure 3.**
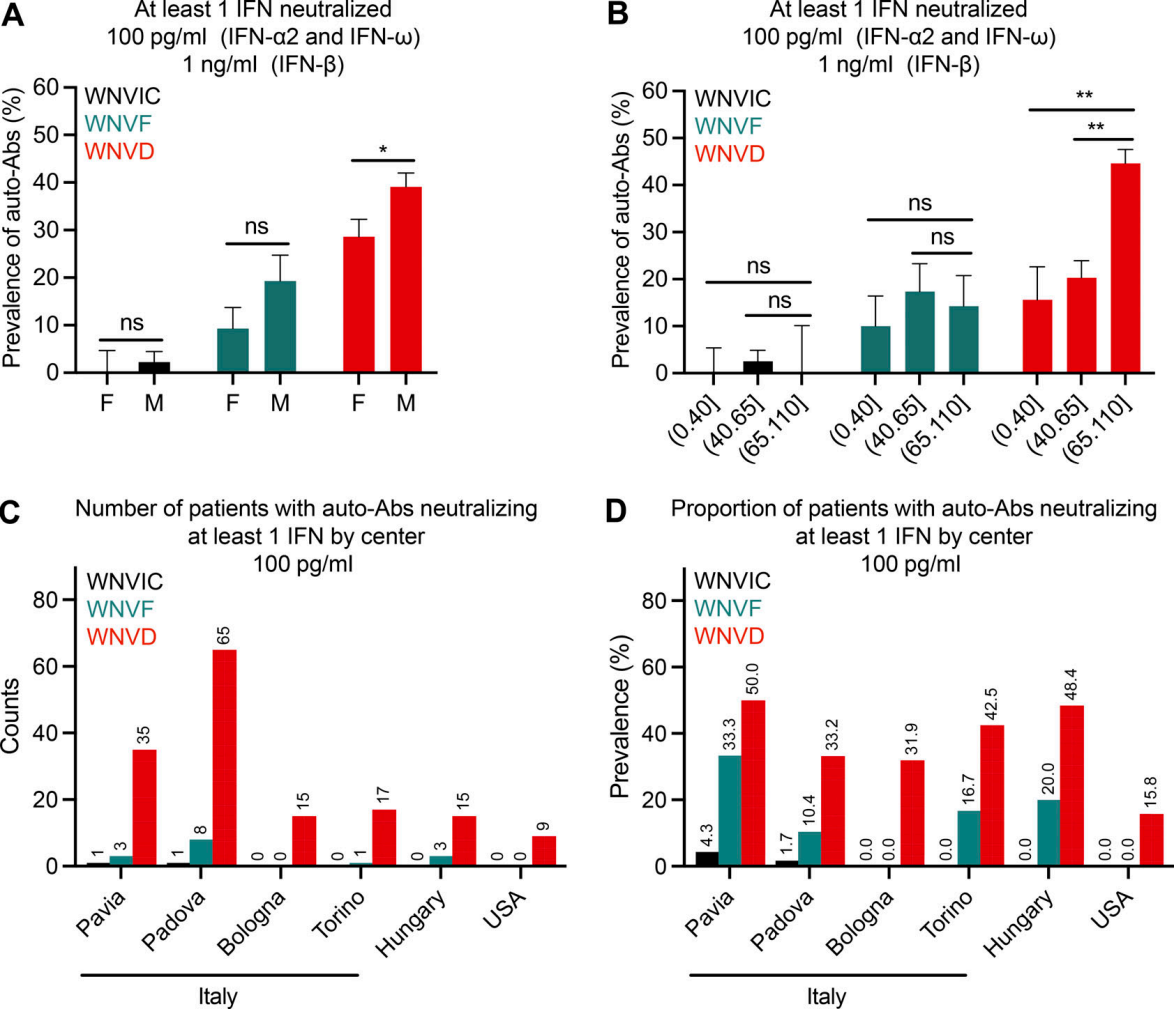
**Proportions of patients with auto-Abs neutralizing type I IFNs by sex, age, and enrolling center. (A)** Prevalence of individuals with auto-Abs neutralizing at least one type I IFN at a concentration of 100 pg/ml (IFN-α2, IFN-ω) or 1 ng/ml (IFN-β) in the three groups of individuals infected with WNV (WNVIC, WNVF, WNVD) by sex. **(B)** Prevalence of individuals with auto-Abs neutralizing at least one type I IFN at a concentration of 100 pg/ml (IFN-α2, IFN-ω) or 1 ng/ml (IFN-β) in the three groups of WNV-infected individuals (WNVIC, WNVF, WNVD) by age class. ns: non-significant; *P *<* 0.05, **P *<* 0.01. **(C and D)** Number (C) and proportion (D) of patients with auto-Abs neutralizing at least one type I IFN by enrolling center.

We then investigated the effects of sex and age on the prevalence of auto-Abs neutralizing low concentrations of IFN-α2 and/or IFN-ω, and/or intermediate concentrations of IFN-β in each disease category. The prevalence of auto-Abs was higher in male subjects than in female subjects in the total cohort (124/428 [29%] vs. 49/235 [21%], OR = 1.55, 95% CI: 1.06–2.26, P = 0.023) and in the WNVD group (112/286 [39%] vs. 44/155 [28%], OR = 1.62, 95% CI: 1.06–2.48, P = 0.024). A similar but non-significant trend was observed in the neuroinvasive disease group (96/232 [41%] vs. 39/116 [34%], OR = 1.39, 95% CI: 0.87–2.22, P = 0.16) and the WNVF group (10/55 [18%] vs. 5/53 [9%], OR = 2.13, 95% CI: 0.68–6.72, P = 0.20; Fig. 3 A; data not shown for the neuroinvasive disease group). The individuals with neutralizing auto-Abs were significantly older than those without such auto-Abs in the total cohort (mean age [SD], 71 [15] yr vs. 60 [18] yr, P = 3.3 × 10^−12^) and in the WNVD group (mean age [SD], 73 yr [14] vs. 65 yr [17], P = 1.8 × 10^−6^). This difference was not observed in the WNVF group (mean age [SD], 56 yr [18] vs. 55 yr [21], P = 0.83). In an analysis in which age was categorized into three classes (≤40, (40–65], and >65 yr), the prevalence of neutralizing auto-Abs was similar in the ≤40 and (40–65] age groups across WNV infection phenotypes (Fig. 3 B). By contrast, the prevalence of neutralizing auto-Abs was higher in subjects >65 yr old than in subjects ≤65 yr old in the WNVD group (125/280, 45% vs. 31/161, 19%, OR = 3.38, 95% CI: 2.14–5.34; P = 1.8 × 10^−7^) and in patients with neuroinvasive disease (125/280, 45% vs. 31/161, 19%, OR = 3.13, 95% CI: 1.86–5.26, P = 1.6 × 10^−5^; Fig. 3 B; data not shown for the neuroinvasive disease group). The much higher prevalence of auto-Abs in WNVD patients over the age of 65 yr, and, to a lesser extent, in male patients, is consistent with the observed effects of age and sex on both the risk of developing WNVD after exposure to WNV (Lindsey et al., 2010; Lindsey et al., 2012; Murray et al., 2009) and the distribution of auto-Abs in the general population (Bastard et al., 2021a). There were no major differences in the results obtained when the six cohorts were analyzed separately (Fig. 3, C and D).

### Auto-Abs neutralizing type I IFNs in the six cohorts

The prevalence of neutralizing auto-Abs in WNVD cases was high in all cohorts, ranging from 16% in the American cohort (57 cases) to 50% in the Pavia cohort (70 cases), with intermediate values of 32% in the Bologna cohort (47 cases), 33% in the largest Padova cohort (196 cases), 43% in the Torino cohort (40 cases), and 48% in the Hungarian cohort (31 cases; Fig. 3, C and D). The prevalence of neutralizing auto-Abs did not differ between outbreak years, with 76/263 (29%) auto-Ab-positive samples in 2018 (41% of the WNVD group) and 83/316 (26%) auto-Ab-positive samples in 2022 (36% of the WNVD group). During the 2022 outbreak in northern Italy, a newly emerged lineage of WNV, WNV-1, circulated together with the endemic WNV-2 lineage and appeared to be associated with a higher risk of neuroinvasive disease (Barzon et al., 2022). In a study of a subset of samples collected at the participating center in Padova in 2022, we found auto-Abs neutralizing type I IFNs in 23/69 (23%) WNVD cases infected with the WNV-1 lineage and in 15/26 (58%) WNVD cases infected with the WNV-2 lineage. This difference was unlikely to be attributable to the infecting strain as we detected neutralizing auto-Abs in 25/89 (28%) WNVD cases in a subset of samples collected at the same center in 2018, when only the WNV-2 lineage was circulating. Thus, being over the age of 65 yr and, to a lesser extent, being male, were the main factors associated with a higher likelihood of anti-type I IFN auto-Ab carriage in individuals with WNVD. These findings are consistent with previous observations concerning the distribution of these auto-Abs in the general population and patients with critical COVID-19 (Bastard et al., 2021a; Manry et al., 2022).

### Risk of WNVD in individuals with auto-Abs against type I IFNs

**Figure 4.**
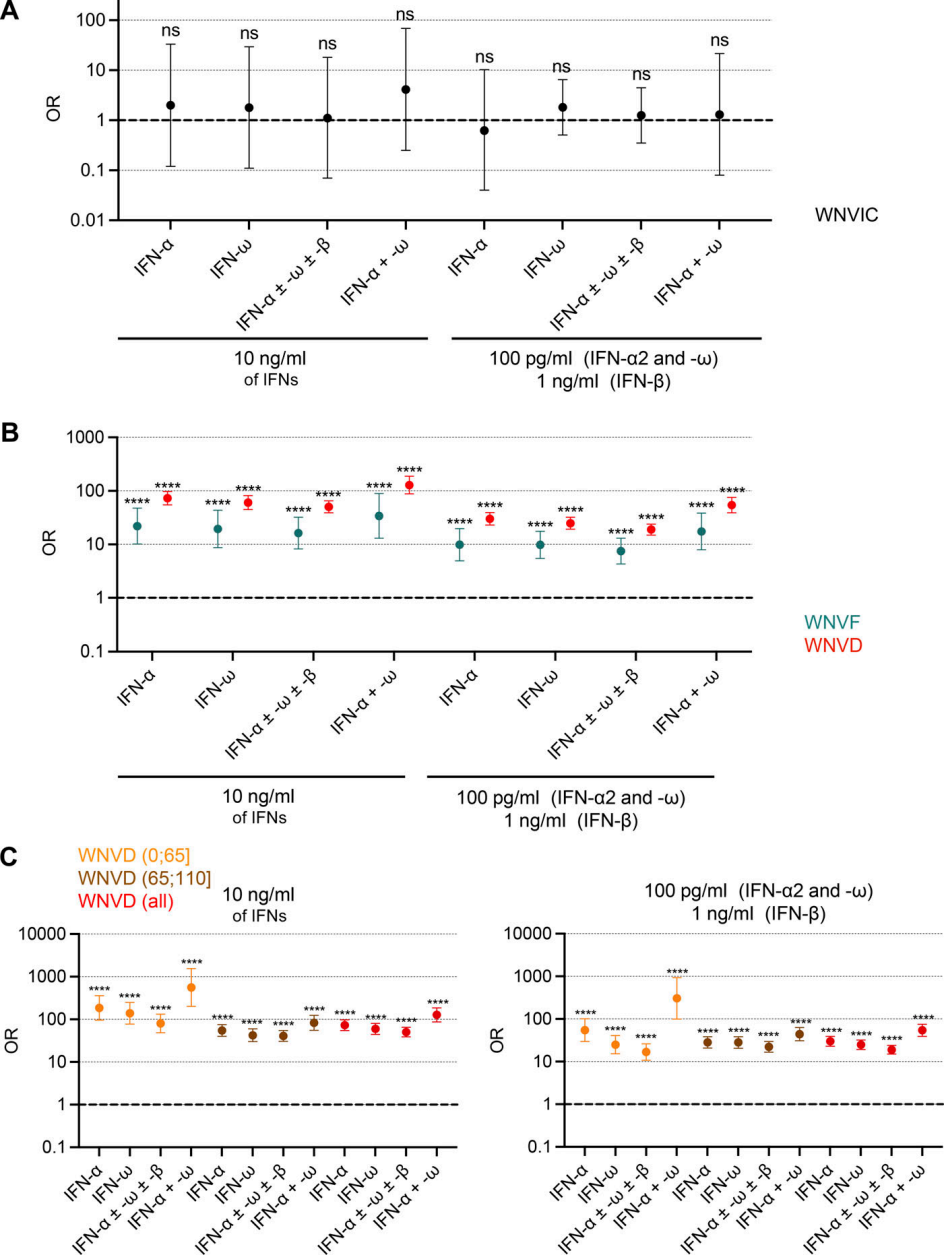
**Enrichment of the WNVF and WNVD groups in auto-Ab-positive individuals relative to the general population. (A)** OR for the presence of auto-Abs in WNVIC relative to the general population, with adjustment for age and sex by Firth’s bias-corrected logistic regression. Firth’s correction can be used to estimate non-zero ORs and finite CIs despite the absence of auto-Ab carriers for some IFN combinations. The horizontal bars indicate the upper and lower limits of the 95% CI. **(B)** OR for the presence of auto-Abs in individuals with WNVF or WNVD relative to the general population, with adjustment for age and sex by logistic regression. The horizontal bars indicate the upper and lower limits of the 95% CI. **(C)** OR for the presence of auto-Abs in individuals with WNVD relative to the general population by age group, with adjustment for age and sex by logistic regression. ORs were calculated separately for patients with WNVD aged ≤65 and >65 yr. The horizontal bars indicate the upper and lower limits of the 95% CI. IFN-α, auto-Abs neutralizing IFN-α2 (regardless of their effects on other IFNs); IFN-ω, auto-Abs neutralizing IFN-ω (regardless of their effects on other IFNs); IFN-α ± ω ± β, auto-Abs neutralizing IFN-α2 and/or IFN-ω and/or IFN-β; IFN-α + ω, auto-Abs neutralizing both IFN-α2 and IFN-ω. ns: non-significant; ****P < 10^−4^.

**Figure S5.**
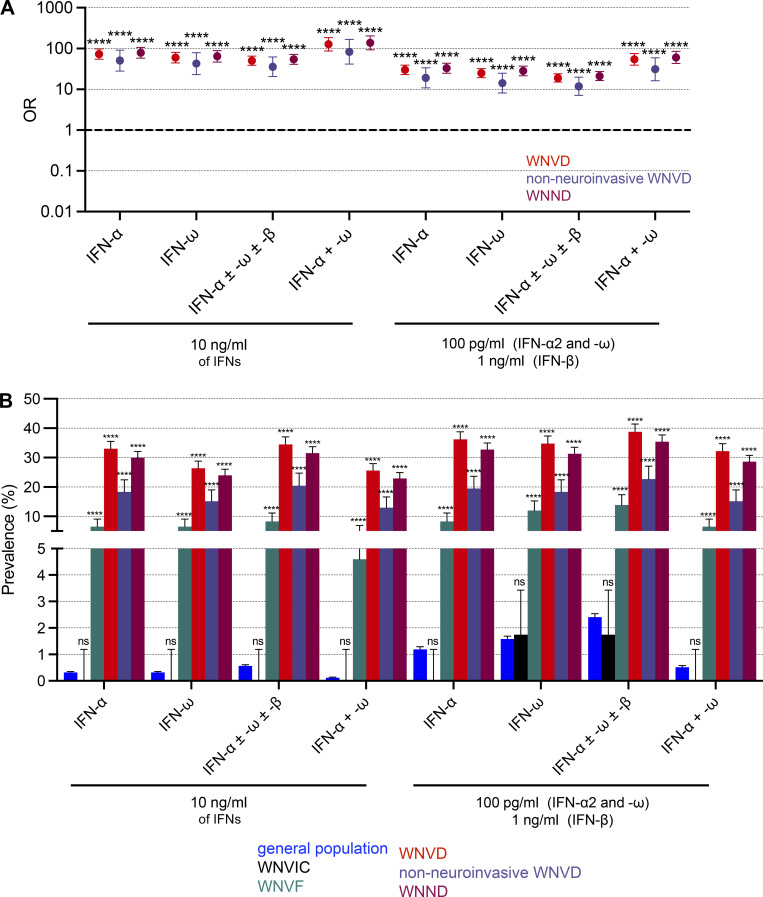
**Enrichment of the WNVD group and the two WNVD subgroups (with and without neuroinvasive-disease) in auto-Ab-positive individuals relative to the general population. (A)** OR for the presence of auto-Abs in the WNVD group and the two WNVD subgroups (WNVD without evidence of neuroinvasive disease, and WNV neuroinvasive disease; WNND) relative to the general population, with adjustment for age and sex by Firth’s bias-corrected logistic regression. Firth’s correction can be used to estimate non-zero ORs and finite confidence intervals despite the absence of auto-Ab carriers for some IFN combinations. The horizontal bars indicate the upper and lower limits of the 95% CI. **(B)** Prevalence of auto-Abs against type I IFNs in the WNVIC, WNVF, and WNVD groups and the two WNVD subgroups (WNVD without evidence of neuroinvasive disease, and WNND) relative to the general population. IFN-α, auto-Abs neutralizing IFN-α2 (regardless of their effects on other IFNs); IFN-ω, auto-Abs neutralizing IFN-ω (regardless of their effects on other IFNs); IFN-α ± ω ± β, auto-Abs neutralizing IFN-α2 and/or IFN-ω and/or IFN-β; IFN-α + ω, auto-Abs neutralizing both IFN-α2 and IFN-ω. ns: non-significant. ****P < 10^−4^.

We estimated the risk of WNVD conferred by the presence and nature of neutralizing auto-Abs by comparing the proportions of subjects with various types or combinations of auto-Abs with the proportions of individuals carrying the corresponding neutralizing auto-Abs in the general population after adjustment for age and sex. General population data were obtained by systematically performing neutralization assays on samples from 34,159 healthy men and women aged 18–100 yr (Bastard et al., 2021a). We found no significant difference between WNVIC and the general population, regardless of the type or combination of auto-Abs considered (Fig. 4 A). Conversely, the prevalence of auto-Abs and of various combinations of these antibodies was significantly higher in all disease groups than in the general population, and it increased with disease severity (Fig. 4 B). The presence of auto-Abs neutralizing at least low concentrations of IFN-α2 and/or IFN-ω was associated with a higher risk of WNVF (OR = 7.3; 95% CI: 4.1–12.8, P = 6.6 × 10^−12^) and WNVD (OR = 19.0; 95% CI: 15.0–24.0, P < 10^−15^; Fig. 4 B and Table 3). The risk was highest for the subgroup of individuals with neuroinvasive disease, the most severe clinical manifestation of WNV infection (OR = 21.1; 95% CI: 16.4–27.1, P < 10^−15^; Fig. S5, A and B; and Table 3). A combination of auto-Abs neutralizing at least low concentrations of both IFN-α2 and IFN-ω further increased the risk of WNVF (OR = 15.8; 95% CI: 6.9–36.3, P = 7 × 10^−11^) and WNVD (OR = 54.5; 95% CI: 39.3–75.6, P < 10^−15^; Fig. 4 B). Again, the risk was highest for neuroinvasive disease (OR = 60.6; 95% CI: 43.1–85.1, P < 10^−15^; Fig. S5, A and B). The risk of clinical disease was even higher in individuals carrying auto-Abs that were also able to neutralize high concentrations (10 ng/ml) of IFN-α2, IFN-β, or IFN-ω. Indeed, the presence of auto-Abs neutralizing high concentrations of IFN-α2 only, IFN-ω only, or combinations of IFN-α2 and/or IFN-β and/or IFN-ω resulted in a 3 to 40 times higher risk of developing WNVF and a 3 to >100 times higher risk of severe WNVD and neuroinvasive disease (Fig. 4 B; Fig. S5, A and B; and Table 3). Individuals with a combination of auto-Abs neutralizing high concentrations (10 ng/ml) of both IFN-α2 and IFN-ω had the highest risk of clinical disease with about a 40-fold increase in the risk of WNVF (OR = 37.0; 95% CI: 13.7–94.6, P = 3.7 × 10^−13^) and a >100-fold increase in the risk of WNVD (OR = 127.4; 95% CI: 87.1–186.4, P < 10^−15^) and neuroinvasive disease (OR = 138.4; 95% CI: 93.3–205.4, P < 10^−15^; Fig. 4 B; and Fig. S5, A and B).

**Table 3.** Risk of WNVF, WNVD, or WNND for subjects carrying auto-Abs neutralizing specific sets of type I IFNs, relative to the general population, with adjustment for age and sex and risk of WNVD by age group


Anti-type I IFN auto-Ab (amount of type I IFN neutralized, in plasma diluted 1:10)	WNV group	OR [95% CI]	P value
Anti-IFN-ω (100 pg/ml)	WNVF	9.3 [5.1–17.1]	4.2 × 10^−13^
Anti-IFN-α2 (100 pg/ml)	WNVF	9.2 [4.4–19.0]	2.3 × 10^−9^
Anti-IFN-α2 (100 pg/ml) and/or anti-IFN-ω (100 pg/ml) and/or anti-IFN-β (10 ng/ml)	WNVF	7.3 [4.1–12.8]	6.6 × 10^−12^
Anti-IFN-α2 (100 pg/ml) and anti-IFN-ω (100 pg/ml)	WNVF	15.8 [6.9–36.3]	7.0 × 10^−11^
Anti-IFN-ω (10 ng/ml)	WNVF	20.4 [9.2–45.7]	1.8 × 10^−13^
Anti-IFN-α2 (10 ng/ml)	WNVF	20.0 [8.8–45.2]	6.5 × 10^−13^
Anti-IFN-α2 (10 ng/ml) and/or anti-IFN-ω (10 ng/ml) and/or anti-IFN-β (10 ng/ml)	WNVF	15.2 [7.4–31.0]	9.1 × 10^−14^
Anti-IFN-α2 (10 ng/ml) and anti-IFN-ω (10 ng/ml)	WNVF	36.0 [13.7–94.6]	3.7 × 10^−13^
Anti-IFN-ω (100 pg/ml)	WNVD (all)	24.9 [19.3–32.2]	<10^−15^
	WNVD ≤ 65 WNVD > 65 WNVD ≤ 70 WNVD > 70	25.0 [15.4–40.6] 28.2 [20.6–38.6] 34.1 [22.5–51.6] 21.9 [15.7–30.7]	<10^−15^ <10^−15^ <10^−15^ <10^−15^
Anti-IFN-α2 (100 pg/ml)	WNVD (all)	30.1 [23.0–39.3]	<10^−15^
	WNVD ≤ 65 WNVD > 65 WNVD ≤ 70 WNVD > 70	54.8 [29.6–101.6] 28.3 [20.9–38.4] 59.3 [36.1–97.4] 22.9 [16.5–31.7]	<10^−15^ <10^−15^ <10^−15^ <10^−15^
Anti-IFN-α2 (100 pg/ml) and/or anti-IFN-ω (100 pg/ml) and/or anti-IFN-β (10 ng/ml)	WNVD (all)	19.0 [15.0–24.0]	<10^−15^
	WNVD ≤ 65 WNVD > 65 WNVD ≤ 70 WNVD > 70	16.8 [10.7–26.3] 22.2 [16.7–29.6] 21.7 [14.9–31.6] 18.5 [13.5–25.2]	<10^−15^ <10^−15^ <10^−15^ <10^−15^
Anti-IFN-α2 (100 pg/ml) and anti-IFN-ω (100 pg/ml)	WNVD (all)	54.5 [39.3–75.6]	<10^−15^
	WNVD ≤ 65 WNVD > 65 WNVD ≤ 70 WNVD > 70	304.6 [99.4–933.3] 43.9 [30.8–62.5] 284.8 [117.1–692.2] 32.6 [22.4–47.4]	<10^−15^ <10^−15^ <10^−15^ <10^−15^
Anti-IFN-ω (10 ng/ml)	WNVD (all)	59.9 [44.4–80.9]	<10^−15^
	WNVD ≤ 65 WNVD > 65 WNVD ≤ 70 WNVD > 70	139.2 [77.6–249.7] 42.4 [30.2–59.6] 141.5 [87.0–230.4] 32.4 [22.5–46.7]	<10^−15^ <10^−15^ <10^−15^ <10^−15^
Anti-IFN-α2 (10 ng/ml)	WNVD (all)	73.2 [54.9–97.6]	<10^−15^
	WNVD ≤ 65 WNVD > 65 WNVD ≤ 70 WNVD > 70	185.6 [96.0–359.0] 54.7 [39.8–75.0] 193.6 [113.8–329.5] 41.2 [29.4–57.8]	<10^−15^ <10^−15^ <10^−15^ <10^−15^
Anti-IFN-α2 (10 ng/ml) and/or anti-IFN-ω (10 ng/ml) and/or anti-IFN-β (10 ng/ml)	WNVD (all)	50.4 [39.0–65.2]	<10^−15^
	WNVD ≤ 65 WNVD > 65 WNVD ≤ 70 WNVD > 70	80.5 [48.6–133.3] 40.9 [30.5–55.0] 88.7 [58.6–134.4] 32.3 [23.5–44.3]	<10^−15^ <10^−15^ <10^−15^ <10^−15^
Anti-IFN-α2 (10 ng/ml) and anti-IFN-ω (10 ng/ml)	WNVD (all)	127.4 [87.1–186.4]	<10^−15^
	WNVD ≤ 65 WNVD > 65 WNVD ≤ 70 WNVD > 70	558.1 [201.7–1,544.1] 82.9 [55.2–124.6] 500.9 [224.5–1,117.5] 59.6 [38.8–91.6]	<10^−15^ <10^−15^ <10^−15^ <10^−15^
Anti-IFN-ω (100 pg/ml)	WNND	28.2 [21.5–37.0]	<10^−15^
Anti-IFN-α2 (100 pg/ml)	WNND	33.0 [24.9–43.9]	<10^−15^
Anti-IFN-α2 (100 pg/ml) and/or anti-IFN-ω (100 pg/ml) and/or anti-IFN-β (10 ng/ml)	WNND	21.1 [16.4–27.1]	<10^−15^
Anti-IFN-α2 (100 pg/ml) and anti-IFN-ω (100 pg/ml)	WNND	60.6 [43.1–85.1]	<10^−15^
Anti-IFN-ω (10 ng/ml)	WNND	64.3 [46.8–88.4]	<10^−15^
Anti-IFN-α2 (10 ng/ml)	WNND	78.8 [58.2–106.8]	<10^−15^
Anti-IFN-α2 (10 ng/ml) and/or anti-IFN-ω (10 ng/ml) and/or anti-IFN-β (10 ng/ml)	WNND	54.3 [41.2–71.5]	<10^−15^
Anti-IFN-α2 (10 ng/ml) and anti-IFN-ω (10 ng/ml)	WNND	138.4 [93.3–205.4]	<10^−15^

WNND: WNV neuroinvasive disease, a subgroup of WNVD. ≤65, >65, ≤70 and >70 indicate age cut-offs. Anti-IFN-ω and anti-IFN-α2 indicate auto-Abs neutralizing IFN-ω or IFN-α2, respectively, regardless of their effects on other IFNs.

### The risk of WNVD due to auto-Abs against type I IFNs is highest in subjects ≤65 yr old

The impact of the auto-Abs on the risk of WNVF and WNVD was greatest in subjects ≤65 yr old. Indeed, the presence of auto-Abs neutralizing high concentrations of both IFN-α2 and IFN-ω increased the risk of WNVF >200-fold (OR = 229.1; 95% CI: 53.4–983.7, P = 2.7 × 10^−13^) and that of WNVD >500-fold (OR = 558.1; 95% CI: 201.7–1,544.1, P < 10^−15^) in subjects ≤65 yr old (Fig. 4 C). In subjects >65 yr old, the same combination of auto-Abs increased the risk of WNVF 12-fold (OR = 11.7; 95% CI: 2.7–51.2, P = 0.001) and the risk of WNVD 80-fold (OR = 82.9; 95% CI: 55.2–124.6, P < 10^−15^; Fig. 4 C). The ORs calculated by age group for all combinations of auto-Abs are reported in Table 3. These results are consistent with previous reports of a greater impact of anti-type I IFN auto-Abs in individuals <70 yr old with critical influenza pneumonia (Zhang et al., 2022b). In our cohort, no major change in the risk was detected if 70 yr was used as the cutoff for age (Table 3). The strong effect of auto-Abs neutralizing type I IFNs on the risk of developing severe disease following exposure to WNV supports the hypothesis that these auto-Abs underlie life-threatening WNVD, and neuroinvasive disease (encephalitis) in particular, in a significant proportion of cases, with their impact being strongest in subjects ≤65 yr old. It also suggests that impaired type I IFN immunity is a major determinant of WNVD.

### The auto-Abs are not induced by WNV

**Figure 5.**
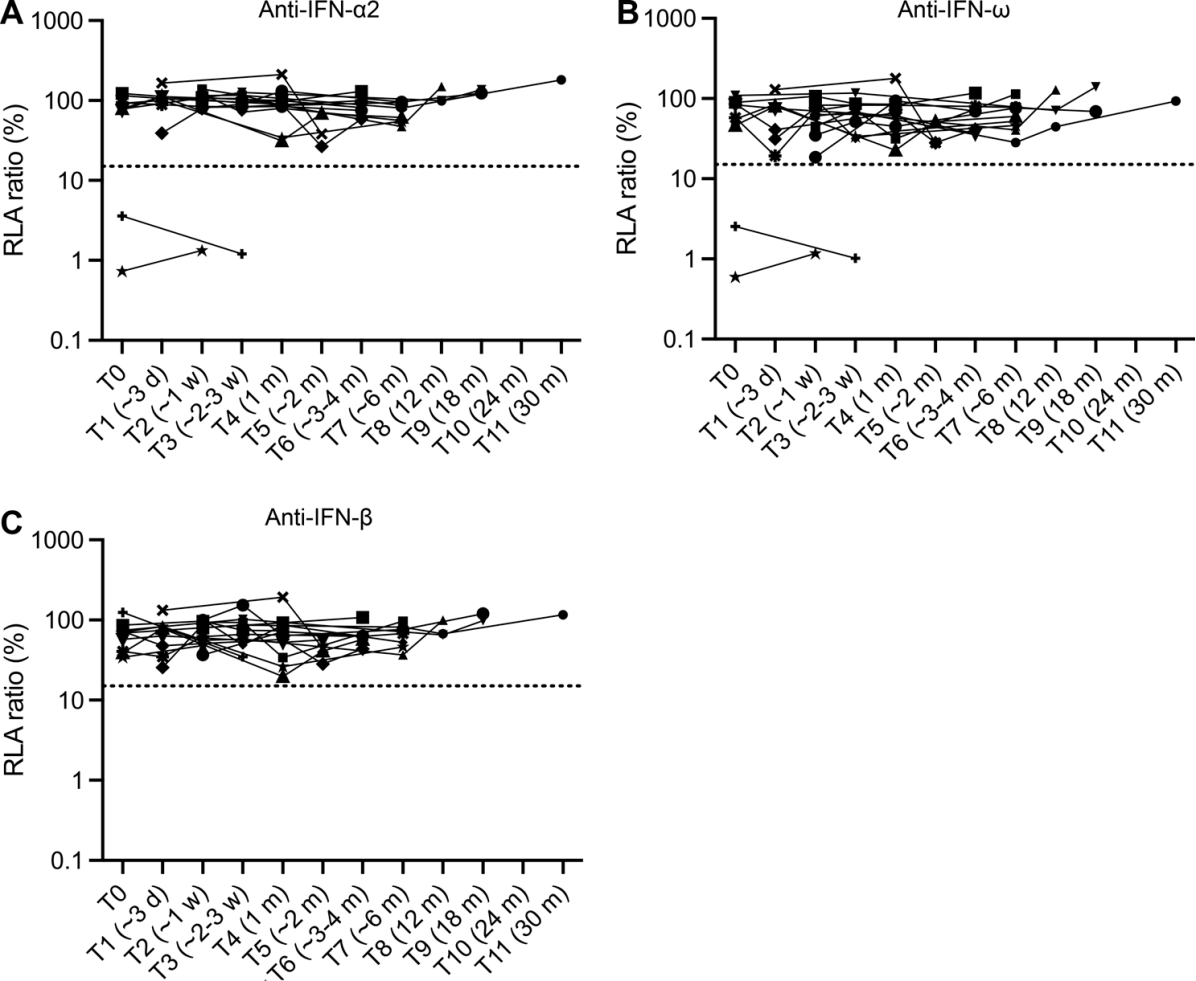
**Testing of auto-Abs against type I IFNs in longitudinal samples. (A–C)** Auto-Abs neutralizing IFN-α2 (A), IFN-ω (B), and IFN-β (C), as determined with the luciferase-based neutralization assay, at different time points after infection. Each sample was tested once with an IFN concentration of 10 ng/ml. The different symbols correspond to different patients testing negative or positive for auto-Abs neutralizing type I IFNs. d: day(s); w: week(s); m: month(s).

In our cohort, 18 samples from auto-Ab-positive patients tested by ELISA were collected within 3 d of the onset of clinical disease (denoted T0) and 61 samples were collected within the first 7 d. The results of these tests attest to the presence of IgG auto-Abs against type I IFNs in the early phases of infection. Moreover, the testing of 109 IgG auto-Ab-positive samples for WNV-specific IgM and IgG and the testing of blood, urine, or CSF for WNV RNA by RT-PCR showed that 4/109 patients tested had detectable WNV but were negative for both IgM and IgG WNV-specific Abs, 1/109 had borderline levels of WNV-IgM and was negative for WNV-IgG, 61/109 had WNV-IgM but were negative for WNV-IgG, 8/109 had IgM and borderline positive values for WNV-IgG, 33/109 had both WNV-specific IgM and IgG, and only 2/109 were negative for IgM and positive for IgG. Thus, 66/109 (61%) IgG auto-Ab-positive individuals had not yet mounted WNV-specific IgG responses at the time of blood sampling, suggesting that the IgG auto-Abs detected were already present before WNV infection. 15 individuals without neutralizing anti-type I IFN auto-Abs at T0 were further tested by ELISA and for the neutralization of high concentrations (10 ng/ml) of IFN-α2, IFN-β, and IFN-ω at multiple time points after infection. We tested 11 asymptomatic or paucisymptomatic WNVIC, 1 WNVF patient, and 3 patients with neuroinvasive disease (1 with meningitis and 2 with encephalitis). We found that no auto-Abs against type I IFNs appeared shortly (first 3 wk) or much later (up to 30 mo) after the diagnosis of WNV infection, regardless of the severity of the clinical manifestations, in the longitudinally tested auto-Ab-negative subjects (Fig. 5, A–C). We also tested longitudinal samples from two individuals with auto-Abs neutralizing high concentrations of both IFN-α2 and IFN-ω, demonstrating the persistence of both auto-Abs at all available timepoints (over ∼3 wk; Fig. 5, A–C). Collectively, these observations suggest that, consistent with previous observations for patients with critical influenza pneumonia, critical COVID-19 pneumonia, or YFV-17D clinical disease (Bastard et al., 2020; Bastard et al., 2021a; Bastard et al., 2021b; Zhang et al., 2022b), anti-type I IFN auto-Abs are not transient and are not induced by WNV infection.

### Auto-Abs neutralizing IFN-α2, -β, and/or -ω in the CSF of patients

**Figure 6.**
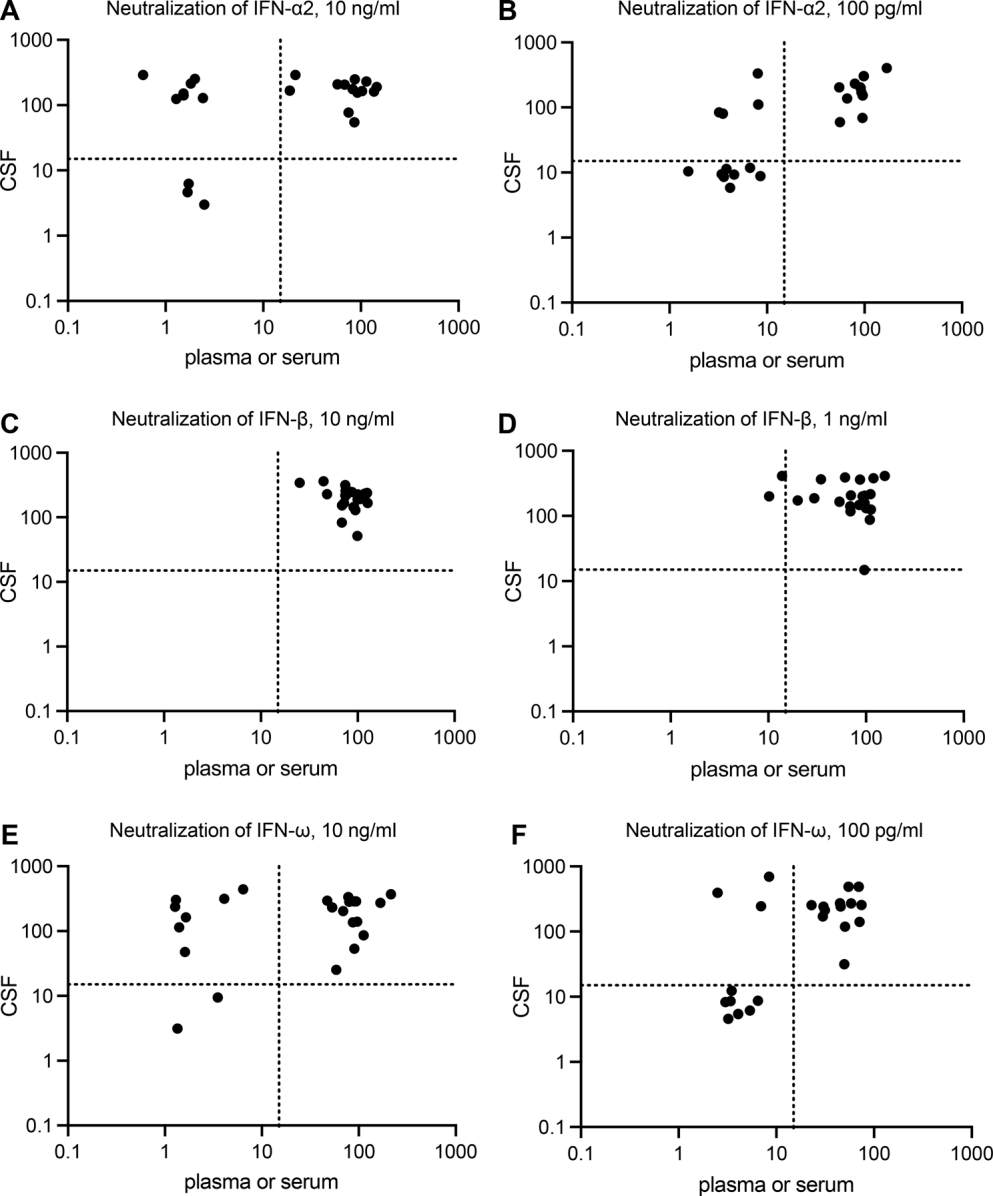
**Auto-Abs in the CSF of patients with WNV neuroinvasive disease. (A–F)** Luciferase-based neutralization assay to detect auto-Abs neutralizing IFN-α2 (A and B for the neutralization of 10 ng/ml and 100 pg/ml, respectively), IFN-β (C and D for the neutralization of 10 ng/ml and 100 pg/ml, respectively), and IFN-ω (E and F for the neutralization of 10 ng/ml and 100 pg/ml, respectively) in the CSF of patients with WNV neuroinvasive disease (y axis). The neutralizing activity of the patient’s serum or plasma is reported on the x axis. CSF samples were diluted 1:10. HEK293T cells were transfected with (1) a plasmid containing the firefly luciferase gene under the control of an ISRE-containing promotor and (2) a plasmid containing the *Renilla* luciferase gene. The cells were then treated with type I IFNs, and relative luciferase activity (RLA) was calculated by normalizing firefly luciferase activity against *Renilla* luciferase activity. An RLA <15% of the value for the mock treatment was considered to correspond to the neutralizing activity of the CSF (dotted line). Each sample was tested once.

CSF samples were available for 23 patients with WNV neuroinvasive disease, collected at the onset of clinical signs along with the serum samples, as part of the diagnostic procedure in cases of suspected infectious encephalitis. We tested 1:10 dilutions of CSF samples from these subjects for the neutralization of high (10 ng/ml) or low (100 pg/ml) concentrations of IFN-α2 and/or IFN-ω, and/or high (10 ng/ml) or intermediate (1 ng/ml) concentrations of IFN-β. We found auto-Abs neutralizing high concentrations of both IFN-α2 and IFN-ω in 1/23 CSF samples, high concentrations of IFN-α2 only in 2/23 CSF samples, and high concentrations of IFN-ω only in 1/23 CSF samples. At the more physiological concentration of 100 pg/ml, we found that 1/23 CSF samples neutralized IFN-α2 only, 2/23 CSF samples neutralized IFN-ω only, and 5/23 samples neutralized both IFN-α2 and IFN-ω. No CSF sample neutralized high or intermediate concentrations of IFN-β (Fig. 6, A–F). We found auto-Abs neutralizing at least low concentrations of IFN-α2 and/or IFN-ω in the CSF of 8/23 (35%) subjects with WNV neuroinvasive disease. All subjects with auto-Abs neutralizing high or low concentrations of IFN-α2 and/or IFN-ω in the CSF also had auto-Abs neutralizing high concentrations of the respective cytokine in serum or plasma samples, and none of the individuals without circulating auto-Abs had auto-Abs in their CSF. Conversely, among the 23 subjects tested for auto-Abs in the CSF, 6/12 subjects with auto-Abs neutralizing at least low concentrations of IFN-α2 in serum (4/10 neutralizing high concentrations of IFN-α2 in serum), 3/10 with auto-Abs neutralizing at least low concentrations of IFN-ω in serum (1/9 neutralizing high concentrations of IFN-ω in serum), and 2/2 with auto-Abs neutralizing intermediate concentrations of IFN-β displayed no detectable neutralizing activity against the corresponding cytokines in the CSF (Fig. 6, A–F). Thus, among the 12/23 individuals with WNV neuroinvasive disease carrying auto-Abs neutralizing at least low concentrations of IFN-α2 and/or IFN-ω in serum or plasma, 8/12 (67%) also had auto-Abs neutralizing the corresponding cytokines in the CSF. It is possible that more sensitive assays might detect the presence of neutralizing auto-Abs in the additional 4/12 patients testing negative in the experimental conditions used here.

### Antibodies against WNV in the CSF of patients

28 individuals from our cohort, with or without serum anti-type I IFN auto-Abs, including 7 of the 8 individuals with auto-Abs in CSF, had previously undergone testing of both serum and CSF for WNV-specific IgM and IgG. The available data for these paired samples show that 12/28 (43%) patients had WNV-specific IgM in serum and 2/8 (25%) patients with WNV-specific IgG in serum also had the same Abs in their CSF. We then focused on the seven individuals with auto-Abs neutralizing IFN-α2 and/or IFN-ω in both serum and CSF. All seven individuals had WNV-specific IgM in serum, 1/7 also tested positive and another one yielded borderline results for WNV-specific IgG in serum. By contrast, only 2/7 CSF samples tested positive and 1/7 was borderline for WNV-specific IgM, whereas 4/7 tested negative for WNV-specific IgM in CSF. Similarly, all CSF samples were negative for WNV-specific IgG. These data suggest that auto-Abs against type I IFNs are present in the CSF before the arrival of newly produced, virus-induced, WNV-specific IgM or IgG, at least in the subjects tested. The small number of CSF samples available for the neutralization assay precluded additional analyses and assessments of the correlation between the presence of anti-type I IFN auto-Abs in the CSF and disease severity. Moreover, CSF samples are not routinely collected from individuals hospitalized for severe WNVD without evidence of neuroinvasive disease, precluding comparisons of CSF neutralization data from individuals with and without neuroinvasive disease. Overall, our findings demonstrate the presence of auto-Abs neutralizing IFN-α2 and/or IFN-ω in the CSF of at least ∼35% of individuals with WNV neuroinvasive disease (at least ∼70% of individuals with neuroinvasive disease and circulating auto-Abs) and suggest that the presence of these auto-Abs in the CSF precedes the development of WNV neuroinvasive disease.

### The auto-Abs neutralize the protective effect of IFN-α2 against WNV

**Figure 7.**
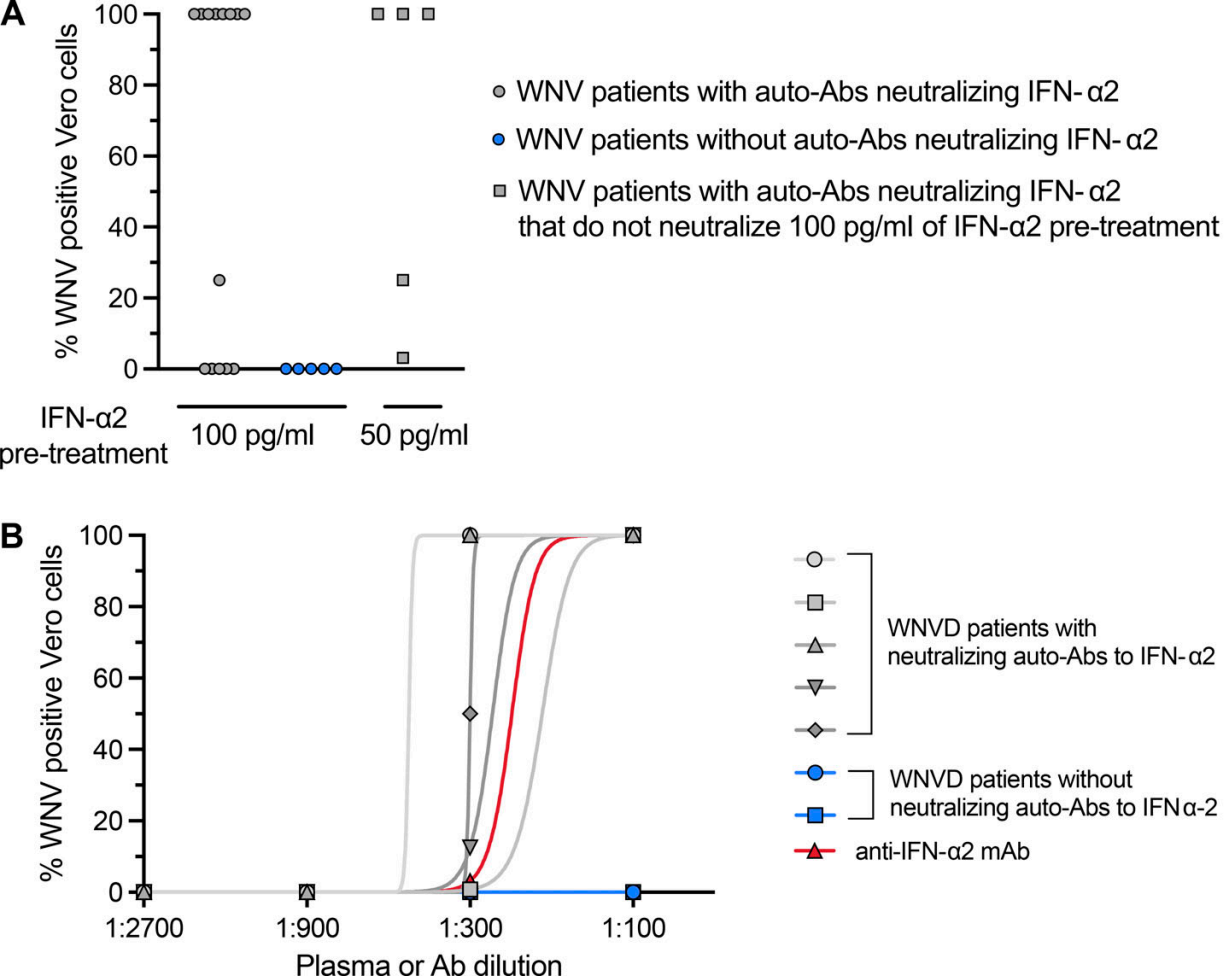
**WNV infection and IFN treatment in Vero-E6 cells. (A)** WNV infection rate, as assessed based on the virus-induced CPE 48 h after infection, in Vero cells treated with various concentrations of IFN-α2 in the presence of plasma from patients with WNVD and neutralizing auto-Abs against IFN-α2 (*n* = 14), or plasma from patients with WNVD without auto-Abs neutralizing type I IFNs (*n* = 5). All the experiments involving the infection of Vero cells with WNV and all the WNV titration experiments were performed in triplicate. **(B)** Enhanced WNV replication, despite the presence of IFN-α2, in the presence of plasma from patients with auto-Abs against IFN-α2. WNV replication, assessed 48 h after infection, in Vero cells treated with IFN-α2 in the presence of plasma from patients with WNVD and neutralizing auto-Abs against IFN-α2 (*n* = 5) or plasma from patients with WNVD without auto-Abs neutralizing type I IFNs (*n* = 2) at different dilutions, or a commercial anti-IFN-α2 monoclonal antibody. All the experiments involving the infection of Vero cells with WNV and WNV titration experiments were performed in triplicate.

Finally, we tested the hypothesis that auto-Abs against type I IFNs impair the protective antiviral functions of type I IFNs in Vero cells infected with WNV. We first confirmed that adding IFN-α2 (100 or 50 pg/ml) before, but not after infection with WNV strain 3B2, protected Vero E6 cells from the cytopathic effect of WNV (data not shown). We then subjected Vero cells to pretreatment with IFN-α2 at a concentration of 100 or 50 pg/ml 24 h before infection in the presence or absence of serum with (*n* = 14) or without (*n* = 5) auto-Abs at a dilution of 1:10 or 1:100 using a previously titrated 50% tissue culture infectious dose (TCID_50_) of WNV diluted 1:100. Under these conditions, 9/14 sera with auto-Abs (at a dilution of 1:100) neutralized IFN-α2 at concentrations of at least 100 pg/ml and the other 5/14 sera (at a dilution of 1:10) with auto-Abs neutralizing IFN-α2 at concentrations of at least 50 pg/ml, whereas none of those without auto-Abs blocked the protective function of IFN-α2 (Fig. 7 A). As a means of excluding the possibility that the simultaneous presence in patient serum of anti-WNV IgM or IgG produced during the acute phase of infection (WNV-NTAbs), itself conferred protection against WNV in vitro, we confirmed that the pretreatment of Vero E6 cells with all the 19 serum samples at a dilution of 1:100 or 1:10 did not confer protection against WNV infection in vitro (data not shown). In the same experimental conditions, we tested threefold serial dilutions of the 1:100 dilution of patient serum to titrate the ability of the serum to neutralize IFN-α2 at a concentration of 50 pg/ml. We found that all five serum samples with auto-Abs tested, unlike the two serum samples without auto-Abs tested, were able to block the antiviral functions of IFN-α2 at dilutions of up to 1:300 (Fig. 7 B). These experiments show that the auto-Abs against type I IFNs found in patients hospitalized for life-threatening WNVD block the protective antiviral activity of IFN-α2 (and probably IFN-β and IFN-ω) against WNV in vitro in cells, allowing unrestricted viral replication.

## Discussion

We show that auto-Abs neutralizing IFN-α and/or IFN-ω account for at least a third of the 441 hospitalizations for life-threatening WNVD in six cohorts from the EU and the USA. These findings suggest that insufficient type I IFN immunity may be a general mechanism of WNV encephalitis. This proportion is similar to that previously reported for a small series of eight patients with adverse reactions to another flavivirus, the YFV-17D live attenuated vaccine (Bastard et al., 2021b). It is, however, higher than that reported for critical COVID-19, influenza, and MERS pneumonia (∼15, ∼5, and 25%, respectively; Bastard et al., 2020; Bastard et al., 2021a; Zhang et al., 2022b; Busnadiego et al., 2022; Hale, 2023; Puel et al., 2022). It is unknown whether these or other unusually severe viral diseases have been observed in patients with WNVD and auto-Abs against type I IFNs, who should be followed longitudinally. Our studies of WNV and YFV-17D suggest that immunity to flaviviruses, which are injected through the skin and directly into the bloodstream by their vectors (Styer et al., 2007), is strongly dependent on the 12 IFN-α subtypes (encoded by 13 loci) and single IFN-ω circulating in the blood (IFN-β being ubiquitous, short-lived, high-affinity, and autocrine; IFN-κ cutaneous; and IFN-ε reproductive). It is tempting to speculate that the 13 subtypes corresponding to IFN-α and IFN-ω, which differ from other type I IFNs in being abundantly secreted into the blood, by plasmacytoid dendritic cells (pDCs) in particular, normally prevent diverse circulating neurotropic viruses from crossing the blood-brain barrier. The testing of cohorts of patients with conditions triggered by arboviruses, including other flaviviruses, such as dengue, yellow fever, tick-borne encephalitis, Japanese encephalitis, and Zika encephalitis, as well as alphaviruses, such as Chikungunya and Ross River virus, is now warranted.

Our findings already have clinical implications. Given the prevalence of auto-Abs against type I IFNs in the general population and the associated risk of life-threatening WNVD, it is probably advisable to test for these antibodies in the general population living in areas in which WNV is endemic, particularly in elderly individuals and those with autoimmune conditions associated with the development of these auto-Abs. It is probably no coincidence that WNVD has been described in a child with autoimmune Addison disease before immunosuppression (Messacar et al., 2014) and in a patient with thymoma (Moen et al., 2022). Given the risk of severe viral diseases other than WNVD, more generalized screening of populations at risk of producing auto-Abs against type I IFNs could be extended to areas in which WNV is not endemic. Individuals with auto-Abs against type I IFNs may benefit from specific preventive measures (e.g., repeat vaccination against COVID-19 and influenza, protection against mosquitoes, contraindication of vaccination with live-attenuated YFV-17D, and travel advice). Patients with WNVD, with or without auto-Abs against type I IFNs, may also benefit from host-directed therapy (Langelier and Vinh, 2022). One patient with autosomal dominant (AD) GATA2 deficiency and WNV encephalitis was recently reported to benefit from IFN-α2 therapy (Rosa et al., 2019). This genetic condition impairs the development of circulating type I IFN-producing pDCs, thereby conferring a predisposition to several severe viral infections (Oleaga-Quintas et al., 2021; Sologuren et al., 2018). Trials of IFN-α2 therapy in patients with WNVD without auto-Abs against IFN-α2 should be considered. Similar treatment options could be envisaged in patients with auto-Abs against type I IFNs, including IFN-β therapy in patients with auto-Abs against IFN-α2 and/or IFN-ω but not IFN-β (Vinh et al., 2021).

## Materials and methods

### Patients

We enrolled an international cohort of 663 individuals aged 9–99 yr with documented WNV infection, 64.5% of whom were male and 35.5% female, living in Italy, Hungary, or the USA (Fig. S1). Written informed consent was obtained in the country of residence of each patient in accordance with local regulations and with institutional review board (IRB) approval. WNV infection was diagnosed on the basis of the serological demonstration of WNV-specific IgM or seroconversion to IgG, WNV neutralization assays (Percivalle et al., 2020), and/or RT-PCR on serum, plasma, or CSF samples. Individuals were stratified according to the presence and/or severity of clinical manifestations, as defined by the need for hospitalization. Life-threatening WNVD was defined as WNV infection requiring hospitalization. WNV fever (WNVF) was defined as WNV infection not requiring hospitalization in patients reporting a febrile illness requiring outpatient care. The WNVIC were blood donors with documented WNV infection, diagnosed on the basis of the detection of WNV RNA in blood during screening at the time of blood donation, who remained asymptomatic or paucisymptomatic (headache) at follow-up. WNVD patients included individuals with confirmed neurological disease (WNV neuroinvasive disease) and individuals without clinical evidence of neuroinvasive disease. The individuals in the neuroinvasive disease group were reported to have encephalitis (fever, acute signs of central or peripheral neurologic dysfunction, including altered mental status and neurological deficits), meningitis (fever, pleocytosis, headache, nuchal rigidity), acute flaccid paralysis (poliomyelitis-like syndrome or Guillain-Barré-like syndrome), or other neurological syndromes. The experiments were conducted in Italy, France, and the USA in accordance with local regulations and guidance from the Italian national data protection authority, the French Ethics Committee (Comité de Protection des Personnes), the French National Agency for Medicine and Health Product Safety, the Institut National de la Santé et de la Recherche Médicale in Paris, France, and with the approval of the IRB of the Italian institutions (San Matteo Research Hospital in Pavia, the University Hospital of Padova, and the University Hospital of Bologna), the National Public Health Center in Budapest, and The Rockefeller University in New York, USA, respectively.

### ELISA

ELISAs were performed as previously described (Puel et al., 2008). In brief, 96-well ELISA plates (MaxiSorp; Thermo Fisher Scientific) were coated by overnight incubation at 4°C with 1 μg/ml rhIFN-α (ref. number 130-108-984; Miltenyi Biotec), rhIFN-ω (ref. number 300-02J; Peprotech), or rhIFN-β (ref. number 300-02BC; Peprotech). Plates were then washed (PBS/0.005% Tween), blocked by incubation with the same buffer supplemented with 2% BSA, washed, and incubated with 1:50 dilutions of plasma samples from the patients or controls for 2 h at room temperature (or with specific mAbs as positive controls). Each sample was tested once. Plates were thoroughly washed (PBS/0.005% Tween). Horseradish peroxidase (HRP)–conjugated Fc-specific IgG fractions from polyclonal goat antiserum against human IgG (Nordic Immunological Laboratories) were added to a final concentration of 1 μg/ml. Plates were incubated for 1 h at room temperature and washed. Substrate was added and OD was measured. All the incubation steps were performed with gentle shaking (600 rpm).

### Gyros

Recombinant human (rh)IFN-α2 (ref. number 130-108-984; Miltenyi Biotec) was first biotinylated with EZ-Link Sulfo-NHS-LC-Biotin (cat. number A39257; Thermo Fisher Scientific), according to the manufacturer’s instructions, with a biotin-to-protein molar ratio of 1:12. Biotinylated rhIFN-α2 was used as a capture reagent at a concentration of 30 µg/ml. The detection reagent contained an Alexa Fluor 647 goat anti-human IgG secondary antibody (ref. number A21445; Thermo Fisher Scientific) diluted in Rexxip F (ref. number P0004825; Gyros Protein Technologies; 1/500 dilution of the 2 mg/ml stock to yield a final concentration of 4 µg/ml). PBS-T 0.01% buffer and Gyros Wash buffer (ref. number P0020087; Gyros Protein Technologies) were prepared according to the manufacturer’s instructions. Plasma or serum samples were then diluted 1/100 in PBS-T 0.01% and tested with the Bioaffy 1000 CD (ref. number P0004253; Gyros Protein Technologies) and the Gyrolab X-Pand (ref. number P0020520; Gyros Protein Technologies). Cleaning cycles were performed in 20% ethanol.

### Luciferase reporter assay

The blocking activity of anti-IFN-α2, anti-IFN-ω, and anti-IFN-β auto-Abs was determined with a reporter luciferase activity. Briefly, HEK293T cells were transfected with a plasmid containing the firefly luciferase gene under the control of the human *ISRE* promoter in the pGL4.45 backbone and a plasmid constitutively expressing *Renilla* luciferase for normalization (pRL-SV40). Cells were transfected in the presence of the X-tremeGene9 transfection reagent (ref. number 6365779001; Sigma-Aldrich) for 24 h. Cells in Dulbecco’s modified Eagle medium (DMEM; Thermo Fisher Scientific) supplemented with 2% fetal calf serum (FCS) and 10% healthy control or patient serum/plasma (after inactivation at 56°C, for 20 min) were either left unstimulated or were stimulated with rhIFN-α2 (ref. number 130-108-984 [not glycosylated]; Miltenyi Biotech or ref. number H6041-10UG [glycosylated]; Merck), rhIFN-ω (ref. number 300-02J [not glycosylated]; Peprotech or TP721113 [glycosylated]; Origene) at 10 ng/ml or 100 pg/ml (not glycosylated) or 1 ng/ml (glycosylated), or rhIFN-β (ref. number 300-02BC [glycosylated]; Peprotech) at 10 or 1 ng/ml for 16 h at 37°C. Finally, cells were lysed for 20 min at room temperature and luciferase levels were measured with the Dual-Luciferase Reporter 1000 assay system (ref. number E1980; Promega) according to the manufacturer’s protocol. Luminescence intensity was measured with a VICTOR-X Multilabel Plate Reader (PerkinElmer Life Sciences). Firefly luciferase activity values were normalized against *Renilla* luciferase activity values. These values were then normalized against the median induction level for non-neutralizing samples and expressed as a percentage. Samples were considered neutralizing if luciferase induction, normalized against *Renilla* luciferase activity, was below 15% of the median values for controls tested the same day.

### WNV infection and IFN treatment in Vero-E6 cells: WNV-NTAbs Microneutralization Assay

All the sera used on Vero E6 cells were tested with the WNV-NTAbs Microneutralization Assay for the detection of WNV-neutralizing antibodies (NTAbs) and determination of their titers, as previously described (Percivalle et al., 2020). Briefly, 50 µl of fourfold (1:10 to 1:640) serially diluted serum samples from patients with or without auto-Abs was added, in duplicate, to a flat-bottomed tissue culture microtiter plate (COSTAR, 13 Corning Incorporated) together with 50 µl (100 TCID_50_) WNV and incubated for 1 h at 33°C under an atmosphere containing 5% CO_2_. Vero E6 cells (VERO C1008 [Vero 76, clone E6, Vero E6]; ATCC CRL-1586; 3 × 10^4^ in 50 µl per well) were then added. After 96 h, the plates were scored for cytopathic effect (CPE) and the neutralizing Ab titer was calculated as the 90% inhibitory concentration (IC_90_), defined as the concentration of Abs able to decrease the percentage of cells infected by 90%. A neutralizing Ab titer <1:10 was considered to be a negative result whereas a titer greater or equal to 1:10 was considered positive. All but one (18/19) of the sera tested also contained WNV-neutralizing Abs. Wild-type WNV (EG101 reference strain; Melnick et al., 1951) was propagated in a BSL-3 laboratory in African green monkey kidney Vero-E6 cells (VERO C1008 [Vero 76, clone E6, Vero E6]; ATCC CRL-1586). Virus stock titers were determined with the Reed-Muench method and are presented as median TCID_50_, as previously described (Percivalle et al., 2020). Vero-E6 cells were used to seed 24-well plates (COSTAR, 13 Corning Incorporated) at a density of 5 × 10^4^ cells/well, with triplicate wells for each set of conditions, including a virus control (no IFN-α2), a cell control (+IFN-α2), and a serum control (serum only, no IFN-α2). The following day, Vero E6 cells were incubated with 100 or 50 pg/ml recombinant human IFN-α2 (11101-2; R&D Systems) for 24 h at 37°C. The plates were then washed with PBS and the cells were infected with 100 TCID_50_ WNV and incubated for 2 h at 37°C. The plates were then washed with PBS to remove the WNV inoculum and fresh medium was added to the wells. After 0, 24, 48, 72, and 96 h, the supernatants were collected and titrated with twofold serial dilutions from 1:1 to 1:128 in 96-well plates for the scoring of WNV CPE on confluent Vero-E6 cells after 5 d of incubation at 37°C. Vero-E6 cells infected as described above were treated with 100 or 50 pg/ml recombinant IFN-α2 in the same experimental conditions. IFN-α2 neutralization by the patients’ serum samples was assessed with Vero-E6 cells used to seed 96-well plates (COSTAR, 13 Corning Incorporated) at a density of 3 × 10^4^ cells/well, with each sample analyzed in triplicate, including a virus control (no IFN-α2), a cell control (with IFN-α2), and a serum control (serum only, no IFN-α2). The following day, a commercial anti–IFN-α2 Ab (21100-1; R&D Systems) or serial threefold dilutions of serum samples were incubated with 100 or 50 pg/ml recombinant IFN-α2 (11101-2; R&D Systems) for 1 h at 37°C (starting dilution: serum samples = 1:100 or 1:10 and anti–IFN-α2 Ab = 1/100). The culture medium was then removed from the cells by aspiration and replaced with the serum/Ab–IFN-α2 mixture. The plates were incubated for 24 h, the serum/Ab–IFN-α2 mixture was then removed, and the plates were washed with PBS to remove any anti-WNV–neutralizing Abs present. Cells were then infected with WNV at 100 TCID_50_ by directly dispensing the inoculum into the wells and incubating for 2 h at 37°C. The plates were then washed with PBS to remove the WNV inoculum and fresh medium was added to the wells. After 48 h, the supernatants were collected and titrated with twofold serial dilutions from 1:1 to 1:128 in 96-well plates for scoring of the WNV CPE on confluent Vero-E6 cells after 5 d of incubation at 37°C.

### Statistical analysis

ORs and P values for the effect of auto-Abs neutralizing each type I IFN in WNV patients relative to healthy individuals from the general population, adjusted for age in three categories (≤40, 41–65, >65 yr) and sex, were estimated by means of logistic regression, as implemented in the glm function of R software. We compared the fit of the model after adjustment for age in three categories, four categories (<40, 41–60, 61–75, >75 yr), or for age as a quantitative variable, and chose the model with the best fit according to Akaike’s information criterion. OR and P values for the effect of auto-Abs on infected controls relative to healthy individuals, adjusted for age and sex, were estimated by means of Firth’s bias-corrected logistic regression, as implemented in the logistf package of R software, due to the absence of auto-Ab carriers for some types of IFN. Where relevant, statistical test results are indicated in the corresponding figures. ns: not significant, *P *<* 0.05, **P *<* 0.01, ***P *<* 0.001, ****P *<* 0.0001.

### Online supplemental material

Fig. S1 shows detailed demographic characteristics of the individuals enrolled in the study. Fig. S2 shows the detection of auto-Abs against type I IFNs by ELISA and Gyros. Fig. S3 shows the detection of auto-Abs neutralizing type I IFNs in the WNVD subgroups and the neutralization of 12 IFN-α2 subtypes. Fig. S4 shows the correlation between the results of luciferase-based neutralization assays with the glycosylated and non-glycosylated IFNs and between the results of neutralization assays and ELISA for detecting type I IFNs. Fig. S5 shows the enrichment of the WNVD group and the two WNVD subgroups (with and without neuroinvasive disease) in auto-Ab-positive individuals relative to the general population.

## Data Availability

All data supporting the findings of this study are available within the main text and supplemental material and from the corresponding authors upon request.
